# In Vitro Reconstitution of Yeast Translation System Capable of Synthesizing Long Polypeptide and Recapitulating Programmed Ribosome Stalling

**DOI:** 10.3390/mps4030045

**Published:** 2021-07-04

**Authors:** Riku Nagai, Yichen Xu, Chang Liu, Ayaka Shimabukuro, Nono Takeuchi-Tomita

**Affiliations:** Department of Computational Biology and Medical Sciences, Graduate School of Frontier Sciences, The University of Tokyo, 5-1-5, Kashiwanoha, Kashiwa-shi, Chiba 277-8562, Japan; nagai-r1@g.ecc.u-tokyo.ac.jp (R.N.); 2966215542@edu.k.u-tokyo.ac.jp (Y.X.); 4793414629@edu.k.u-tokyo.ac.jp (C.L.); 8938900508@edu.k.u-tokyo.ac.jp (A.S.)

**Keywords:** CrPV IGR IRES, in vitro translation, translation elongation, translation termination, yeast

## Abstract

The rates of translation elongation or termination in eukaryotes are modulated through cooperative molecular interactions involving mRNA, the ribosome, aminoacyl- and nascent polypeptidyl-tRNAs, and translation factors. To investigate the molecular mechanisms underlying these processes, we developed an in vitro translation system from yeast, reconstituted with purified translation elongation and termination factors, utilizing CrPV IGR IRES-containing mRNA, which functions in the absence of initiation factors. The system is capable of synthesizing not only short oligopeptides but also long reporter proteins such as nanoluciferase. By setting appropriate translation reaction conditions, such as the Mg^2+^/polyamine concentration, the arrest of translation elongation by known ribosome-stalling sequences (e.g., polyproline and CGA codon repeats) is properly recapitulated in this system. We describe protocols for the preparation of the system components, manipulation of the system, and detection of the translation products. We also mention critical parameters for setting up the translation reaction conditions. This reconstituted translation system not only facilitates biochemical analyses of translation but is also useful for various applications, such as structural and functional studies with the aim of designing drugs that act on eukaryotic ribosomes, and the development of systems for producing novel functional proteins by incorporating unnatural amino acids by eukaryotic ribosomes.

## 1. Introduction

The reconstitution of biological processes is critical for the investigation of the underlying molecular mechanisms. Regarding the translation of mRNA, some reconstituted translation systems have, so far, been developed with bacterial (*Escherichia coli* [1], *Thermus* *thermophilus* [2], *Mycobacterium* [3]), human [4], and mammalian mitochondrial factors [5]. A system from yeast *Saccharomyces cerevisiae* has been long-awaited, as yeast is one of the best-characterized model eukaryotes with a variety of resources. For yeast, the individual steps of translation have been partially reconstituted: initiation [6,7], peptide elongation [8,9,10,11], and termination and ribosome recycling [11,12,13,14]. Combination of these in vitro systems allowed synthesizing oligopeptides, and the direct measurement of the kinetics of translation elongation, by manipulating mRNA coding sequence, tRNA identity and concentration, as well as ribosome and translation factor composition [15]. However, until recently, the translation of a long peptide has not been attempted. This is partly because cap-dependent translation initiation and tRNA aminoacylation are complicated biological processes. Cap-dependent initiation is the most complex stage of translation in yeast, requiring at least 12 initiation factors, with many being composed of several peptides [6]. Moreover, the tRNA aminoacylation process is also highly complex, as 20 different aminoacyl-tRNA synthetases and their cofactors are involved in charging tRNAs with their cognate amino acids [16,17].

We have recently developed an in vitro reconstituted translation system that is capable of synthesizing long peptides [18]. In this system, in order to bypass the complex initiation process, we exploited CrPV IGR IRES (intergenic region internal ribosome entry site sequence from cricket paralysis virus)-containing mRNA, whereby yeast 80S ribosomes initiate in the absence of initiator tRNA or any eukaryotic translation initiation factors [19,20]. The translation elongation process was reconstituted by using yeast elongation factors (eEF1A, eEF2, and the fungal-specific elongation factor eEF3 [15,21,22,23]) and pre-charged aminoacyl-tRNAs. To prepare aminoacyl-tRNAs, a yeast tRNA mixture was charged by yeast S100 extract, which we found to be capable of template-dependent synthesis of long polypeptides. Termination factors (eRF1 and eRF3) and recycling factors (Rli1, Hbs1, and Dom34) were further combined, in order to complete the reconstituted in vitro translation system. By setting appropriate translation reaction conditions, such as the Mg^2+^/polyamine concentration, the arrest of translation elongation by known ribosome-stalling sequences (e.g., polyproline and CGA codon repeats) can be properly recapitulated in this system. This system made it possible to analyze the translation elongation and termination, with greater control over the length and sequence of mRNA and nascent peptides. For example, by utilizing this system, we have examined the role of the eukaryotic translation factor eIF5A and its hypusine modification in translating the polyproline sequence within long open reading frames. We demonstrated that the requirement of the hypusine modification of eIF5A to alleviate the polyproline-mediated ribosome-stalling depends on the location and length of the polyproline motif in the protein [24].

Here, we describe detailed protocols for the preparation of the system components, manipulation of the system, and detection of the translation products. We also mention the translation reaction conditions, in order to recapitulate the programmed ribosome stalling. This reconstituted yeast translation system facilitates biochemical analyses of translation elongation, termination, and ribosome recycling on natural mRNAs, and also provides a framework for studying co-translational events, such as protein folding, targeting, and degradation through the ribosome-associated quality control (RQC) pathway. Moreover, the system is potentially useful for various applications; for example, it is suitable for use in structural and functional studies to design drugs that act on eukaryotic ribosomes, such as antibiotics against fungi. As the system allows for research into the effect of mRNA- or nascent peptide-context on translation termination, it could also be applied for the development of readthrough-inducing agents for treating genetic diseases caused by nonsense mutations. Other applications of this system include the template-dependent synthesis of proteins containing unnatural amino acids by eukaryotic ribosomes, using synthetic tRNAs that have been appropriately charged for example, with ribozymes. In combination with ribosome- or mRNA-display systems, this system could serve as a powerful in vitro screening system for novel functional proteins, such as antimicrobial peptides with cytotoxicity.

## 2. Experimental Design

**Figure 1:**
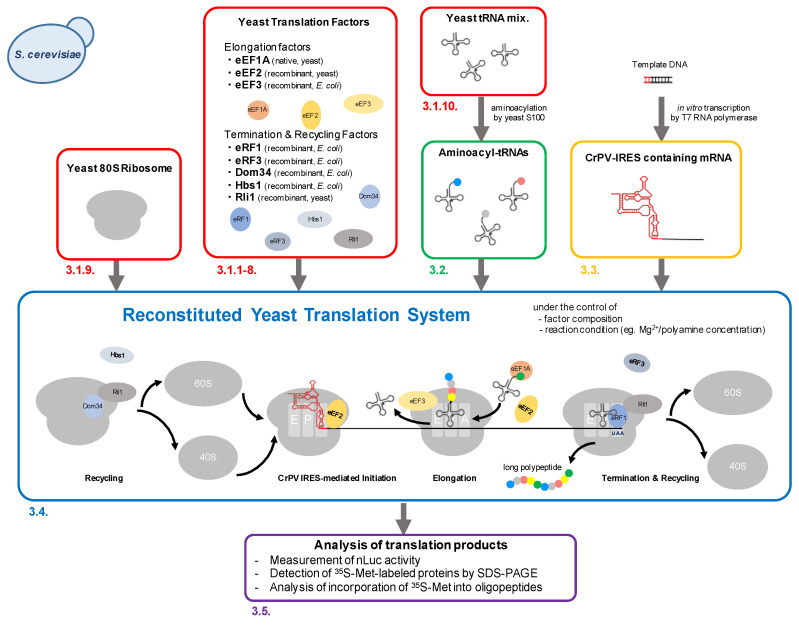
Schematic overview of reconstitution of yeast translation system capable of synthesizing long polypeptides and recapitulating the programmed ribosome stalling. Each procedure is described in the section shown below the rectangle. See the “Experimental Design” section for details.

This protocol describes the reconstitution of the yeast translation system in vitro, which is capable of synthesizing long polypeptides following CrPV-IRES-mediated translation initiation, as well as recapitulating the translation elongation arrest by known downregulating sequences, such as the polyproline sequence. As illustrated in Figure 1, the procedure includes five parts: (i) preparation of yeast translation factors (elongation factors, termination factors, recycling factors, ribosomes, tRNAs); (ii) preparation of aminoacyl-tRNAs by charging yeast crude tRNA mixtures using yeast S100 extract; (iii) preparation of CrPV IRES-containing mRNA by in vitro transcription using T7 RNA polymerase; (iv) the translation reaction; and (v) analysis of the translation products. In the following, we explain the parameters that critically determine the properties of the reconstituted translation system.

*eEF1A concentration*

A high eEF1A concentration—that is, 10- to 20-fold relative to the ribosome concentration—is required for efficient translation reactions, especially to synthesize long polypeptides. For example, the synthesis yield of the reporter protein nanoluciferase (nLuc) using an equivalent amount of eEF1A to ribosomes drops three orders of magnitude from that when using 10-fold eEF1A; that is, the yield of nLuc drops from 0.03 nM to 0.03 pM [18]. We found that such a high eEF1A concentration is important to ensure the dominance of IRES-mediated translation initiation over IRES-independent random internal translation initiation [18]. In the CrPV IRES-mediated translation initiation, the delivery of the first tRNA by eEF1A governs the overall efficiency of initiation [19,25]. Thus, for the long mRNA encoding long polypeptides, ternary complexes (eEF1A•aa-tRNA•GTP) may possibly be consumed by the IRES-independent random internal translation initiation, thereby inhibiting the IRES-dependent translation initiation. Indeed, a high eEF1A concentration is not strictly required when the short mRNA encoding an oligopeptide is translated with the system. The synthesis of oligopeptides using an equivalent amount of eEF1A to ribosomes is reduced to, at most, one-fifth of that using 10-fold eEF1A (i.e., the yield of oligopeptide synthesis drops from 3 nM to 0.6 nM [18]), whereby the required eEF1A amount is roughly proportional to the number of amino acids to be polymerized.

*Magnesium/polyamine concentration*

**Figure 2:**
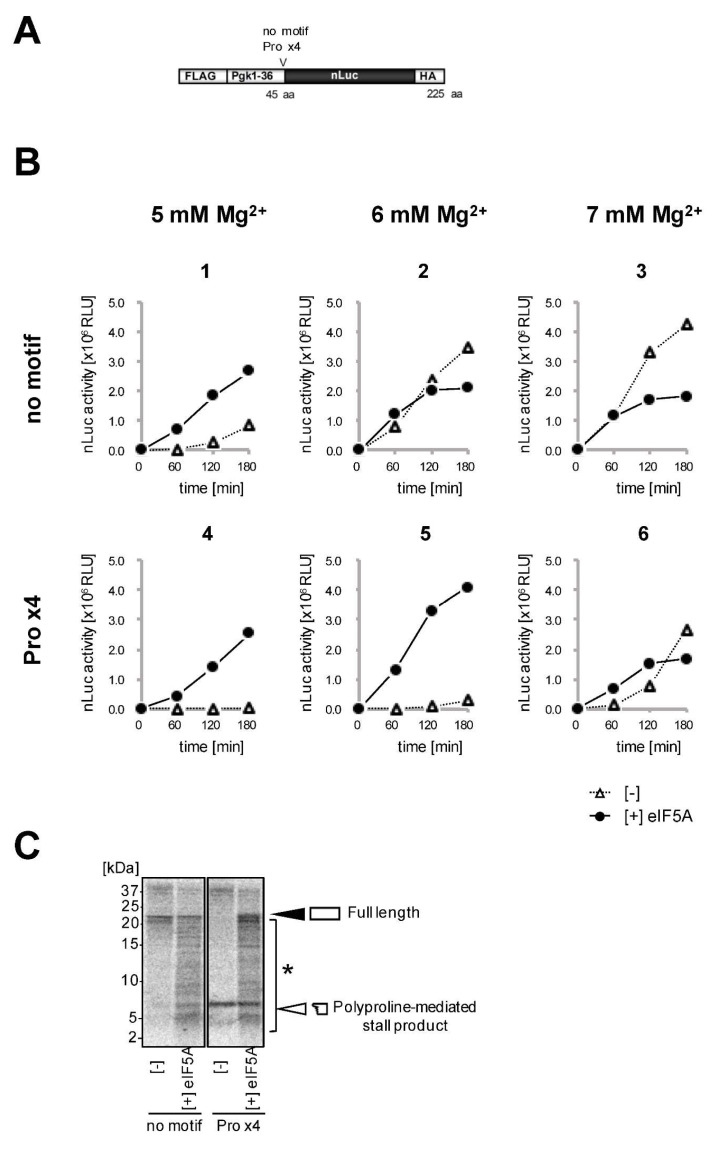
Analysis of translational ability of purified 80S ribosomes and determination of the appropriate Mg^2+^ concentration to recapitulate the polyproline-mediated ribosomal stalling. After the purification of ribosomes, their translational abilities are checked using the indicated mRNAs (see also Table S4). Shown are the examples: (**A**) schematic of the mRNAs; (**B**) translation reactions were performed using mRNA and [^35^S]methionine-labelled aminoacyl-tRNA mix, with 5–7 mM Mg^2+^, 0.25 mM spermidine, and 0 mM spermine. Where indicated, 0.5 μM hypusinated eIF5A protein—equivalent to the amount of ribosomes—was included in the reaction. After incubation for the indicated time period, aliquots were subjected to nLuc assay. [-] (open triangle), no protein; [+] eIF5A (closed circle), eIF5A(hyp). (**C**) After 180-min reaction, the translation products at 6 mM Mg^2+^ (Panels 2 and 5 in (**B**)) were analyzed by Tricine SDS-PAGE. Black triangle, full-length product; open triangle, polyproline-mediated stall product. Asterisk indicates unknown products that are specifically produced in the presence of eIF5A, and may possibly be premature termination products (see text and [24]). For the preparation of eIF5A, see Appendix C.

It is important to set an appropriate Mg^2+^ and polyamine concentrations in the reconstituted yeast translation system, in order to recapitulate polyproline-mediated ribosome stalling. We have previously demonstrated that polyproline arrests translation in a manner similar to ‘intrinsic ribosome destabilization (IRD)’ [24]. IRD is a recently discovered phenomenon in bacteria, where consecutive and proline-intermitted acidic amino acids destabilize the 70S ribosome from within the peptide tunnel and abort translation [26]. Thus, in a yeast reconstituted translation system, under conditions with relatively lower Mg^2+^, polyproline destabilizes both the peptidylpolyproline-tRNA itself and the ribosome, inhibiting the peptidyl transfer reaction. However, under conditions with relatively higher Mg^2+^ (where ribosomes are stable), the disorder of the peptidylpolyproline-tRNA is resolved, consequently alleviating polyproline-mediated ribosome stalling. To analyze polyproline-mediated ribosome stalling or other potential magnesium/polyamine-dependent translation regulation, we usually set the concentration of Mg^2+^ in a range of about 5 to 7 mM, in the presence of 0.25 mM spermidine. The precise Mg^2+^ concentration needs to be determined, depending on the lot of 80S ribosome preparation, as described later (see Figure 2). Ribosome stalling is alleviated and no longer observed at Mg^2+^ concentrations above a specific concentration between 5 and 7 mM.

We commonly use mRNA and pre-charged aa-tRNAs for the translation reaction in the reconstituted translation system (Figure 1). On the other hand, by including a DNA template, T7 RNA polymerase, and/or purified aminoacyl-tRNA synthetases (human aminoacyl-tRNA synthetases [4]) in the system, it is also possible to perform a translation reaction coupled with transcription and/or aminoacylation reaction, which greatly simplifies the experiments and saves time (see Section 3.4); however, care must be taken that the T7 RNA polymerase and aminoacyl-tRNA synthetases consume triphosphate nucleotides which chelate Mg^2+^ during the reaction, such that the control of Mg^2+^ concentration is difficult in such transcription- and aminoacylation-coupled systems.

We prefer polyamine-containing conditions, such as 5 mM Mg^2+^, 0.25 mM spermidine, and 0 mM spermine (designated hereafter as [5/0.25/0]), over polyamine-free conditions, in order to maintain the translation fidelity [27].

### 2.1. Reagents

- HEPES (Dojindo, Tokyo, Japan, Cat. no.: 342-01375)
- Potassium hydroxide (KOH) (Wako, Tokyo, Japan, Cat. no.: 168-21815)
- Tris(hydroxymethyl)aminomethane (Tris) (Wako, Cat. no.: 207-06275)
- Hydrochloric acid (HCl) (Wako, Cat. no.: 080-01066)
- Acetic acid (Wako, Cat. no.: 017-00251)
- Boric acid (Wako, Cat. no.: 021-02195)
- EDTA•2Na (EDTA) (Dojindo, Cat. no.: 345-01865)
- Potassium chloride (KCl) (Wako, Cat. no.: 163-03545)
- Ammonium acetate (Wako, Cat. no.: 019-02835)
- Ammonium chloride (NH_4_Cl) (Wako, Cat. no.: 017-02995)
- Magnesium chloride hexahydrate (MgCl_2_) (Wako, Cat. no.: 135-00165)
- Magnesium acetate tetrahydrate (Mg(OAc)_2_) (Wako, Cat. no.: 130-00095)
- MOPS (Dojindo, Cat. no.: 343-01805)
- Imidazole (Wako, Cat. no.: 095-00015)
- Ammonium sulfate ((NH_4_)_2_SO_4_) (Wako, Cat. no.: 013-03433)
- Sucrose (Wako, Cat. no.: 196-00015)
- Dithiothreitol (DTT) (Wako, Cat. no.: 049-08972)
- 2-Mercaptoethanol (β-Me) (Wako, Cat. no.: 131-14572)
- Phenylmethanesulfonyl fluoride (PMSF) (Sigma-Aldrich, St. Louis, MO, USA, Cat. no.: P7626)
- Isopropyl-β-D-thiogalactopyranoside (IPTG) (Nacalai tesque, Tokyo, Japan, Cat. no.: 19742-94)
- Sodium chloride (NaCl) (Wako, Cat. no.: 191-01665)
- Bacto tryptone (Thermo Fisher Scientific, Waltham, MA, USA, Cat. no.: 211705)
- Bacto yeast extract (Thermo Fisher Scientific, Cat. no.: 212750)
- Hipolypepton (Wako, Cat. no. 392-02115)
- D(+)-Glucose (Wako, Cat. no.: 049-31165)
- D(+)-Raffinose (Wako, Cat. no.: 184-00015)
- D(+)-Galactose (Wako, Cat. no.: 075-00035)
- Difco yeast nitrogen base w/o amino acids (Becton, Dickinson and company, Sparks, MD, USA, Cat. no.: 291940)
- Sodium hydroxide (NaOH) (Wako, Cat. no.: 198-13765)
- Sodium acetate, anhydrous (NaOAc) (Wako, Cat. no.: 192-01075)
- Potassium acetate (KOAc) (Wako, Cat. no.: 160-03175)
- Phenol, granular (Nacalai tesque, Cat. no.: 26728-45)
- 2-Propanol (Wako, Cat. no.: 166-04836)
- Ethanol (Wako, Cat. no.: 057-00451)
- Methanol (Wako, Cat. no.: 137-01823)
- Chloroform (Wako, Cat. no.: 038-02601)
- Isoamyl alcohol (Tokyo chemical industry, Tokyo, Japan, Cat. no.: I0289)
- ISOGEN-LS (Nippon gene, Tokyo, Japan, Cat. no.: 311-02621)
- Formaldehyde solution (Formalin) (Nacalai tesque, Cat. no.: 16223-55)
- Formamide (Nacalai tesque, Cat. no.: 16345-65)
- Complete mini protease inhibitor cocktail tablets, EDTA-free (Sigma-Aldrich, St. Louis, MO, USA, Cat. no.: 11836170001)
- 30 *w/v*% acrylamide/Bis mixed solution (37.5:1) (Wako, Cat. no.: 018-25625)
- Sodium dodecyl sulfate (SDS) (Wako, Cat. no.: 191-07145)
- Ammonium peroxodisulfate (APS) (Wako, Cat. no.: 012-03285)
- *N*,*N*,*N’*,*N’*-tetramethyl-ethylenediamine (TEMED) (Wako, Cat. no.: 205-06313)
- Urea (Wako, Cat. no.: 215-00616)
- CBB R-250 (CBB) (Wako, Cat. no.: 031-17922)
- Xylene cyanole FF (XC) (Sigma-Aldrich, Cat. no.: X4126)
- Bromophenol blue (BPB) (Wako, Cat. no.: 021-02911)
- Lithium dodecyl sulfate (LiDS) (Sigma-Aldrich, Cat. no.: L4632)
- Acrylamide (Wako, Cat. no.: 011-08015)
- *N*,*N’*-methylene-bis(acrylamide)-HG (BIS) (Wako, Cat. no.: 138-08173)
- Bio-Rad Protein Assay Dye Reagent Concentrate (Bio-Rad, Hercules, CA, USA, Cat. no.: 5000006)
- Ethidium bromide solution 10 mg/mL (EtBr) (Nacalai tesque, Cat. no.: 14631-94)
- ATP lithium salt (ATP) (Roche, Cat. no.: 11140965001)
- CTP lithium salt (CTP) (Roche, Cat. no.: 11140922001)
- GTP lithium salt (GTP) (Roche, Cat. no.: 11140957001)
- UTP lithium salt (UTP) (Roche, Cat. no.: 11140949001)
- Creatine phosphate (CP) (Roche, Cat. no.: 10621722001)
- Spermidine (SPD) (Wako, Cat. no.: 197-13833)
- Spermine (SP) (Wako, Cat. no.: 194-09813)
- L-Glutamic acid potassium salt monohydrate (Sigma-Aldrich, Cat. no.: G1501)
- Adenine hydrochloride (Adenine) (Wako, Cat. no.: 016-00802)
- myo-Inositol (L-Inositol) (Wako, Cat. no.: 092-00282)
- p-Aminobenzoic acid (Wako, Cat. no.: 015-02332)
- L-Alanine (Ala) (Wako, Cat. no.: 010-01042)
- L-Arginine (Arg) (Sigma-Aldrich, Cat. no.: A8094)
- L-Asparagine monohydrate (Asn) (Wako, Cat. no.: 019-04812)
- L-Aspartic acid (Asp) (Wako, Cat. no.: 013-04832)
- L(+)-Glutamine (Gln) (Wako, Cat. no.: 074-00522)
- L-Glutamic acid (Glu) (Wako, Cat. no.: 070-00502)
- Glycine (Gly) (Wako, Cat. no.: 073-00732)
- L-Histidine (His) (Sigma-Aldrich, Cat. no.: H6034)
- L(+)-Isoleucine (Ile) (Wako, Cat. no.: 123-00861)
- L-Leucine (Leu) (Wako, Cat. no.: 126-00851)
- L-Lysine (Lys) (Sigma-Aldrich, Cat. no.: L5501)
- L(-)-Phenylalanine (Phe) (Wako, Cat. no.: 163-01301)
- L(-)-Proline (Pro) (Wako, Cat. no.: 163-04601)
- L-Serine (Ser) (Wako, Cat. no.: 191-00401)
- L(-)-Threonine (Thr) (Wako, Cat. no.: 206-01321)
- L-Tryptophan (Trp) (Wako, Cat. no.: 206-03381)
- L-Tyrosine (Tyr) (Wako, Cat. no.: 204-03561)
- L-Valine (Val) (Wako, Cat. no.: 220-00081)
- L-Cysteine (Cys) (Sigma-Aldrich, Cat. no.: C7352)
- L-Methionine (Met) (Wako, Cat. no.: 133-01602)
- Methionine, L-[^35^S]-(Met) (Perkin Elmer, Waltham, MA, USA, Cat. no.: NEG009A)
- Guanidine thiocyanate (Nacalai tesque, Cat. no.: 17345-35)
- Trisodium citrate dihydrate (Wako, Cat. no.: 191-01785)
- N-Lauroylsarcosine sodium salt (Sarcosyl) (Nacalai tesque, Cat. no.: 20117-12)
- Y-PER Yeast Protein Extraction Reagent (Thermo Fisher Scientific, Cat. no.: 78990)
- TWEEN 20 Detergent (Merck, Darmstadt, Germany, Cat. no.: 655205)
- Heparin sodium (Wako, Cat. no.: 081-00136)
- Puromycin dihydrochloride (puromycin•2HCl) (Wako, Cat. no.: 166-23153)
- Glycerol (Wako, Cat. no.: 075-00611)
- KOD-Plus-Neo (Toyobo, Tokyo, Japan, Cat. no.: KOD-401)
- *Dpn* I (Takara, Tokyo, Japan, Cat. no.: 1235A)
- 10× loading buffer (Takara, Cat. no.: 9157)
- Agarose L03 TAKARA (Takara, Cat. no.: 5003)
- Recombinant RNase Inhibitor (Takara, Cat. no.: 2313A)
- TURBO DNase (Thermo Fisher Scientific, Cat. no.: AM2238)
- RNase A (Macherey-Nagel, Düren, Germany, Cat. no.: 740505.05)
- RNase-free water (generated by an ultrapure water system)

### 2.2. Equipment

- AKTA explorer (Cytiva, Tokyo, Japan)
- Ultracentrifuge (Beckman coulter, Fullerton, CA, USA, Cat. no.: Optima LE-80K)
- Type 45 Ti Rotor (Beckman coulter, Cat. no.: 339160)
- Type 70 Ti Rotor (Beckman coulter, Cat. no.: 337922)
- Ultracentrifuge tube for 45 Ti, 70 mL (Beckman coulter, Cat. no.: 355622)
- Ultracentrifuge tube for 70 Ti, 26.3 mL (Beckman coulter, Cat. no.: 355618)
- Mortar Mill RM200 (Retsch, Rheinische, Germany)
- Digital Sonifier 450 (Branson, Danbury, CT, USA)
- BAS5000 (Fujifilm, Tokyo, Japan)
- Scintillation counter, LSC-6100 (ALOKA, Tokyo, Japan)
- Wizard SV Gel and PCR Clean-Up System (Promega, Madison, WI, USA, Cat. no.: A9281)
- Micro Bio-Spin columns P-30, Tris (Bio-Rad, Cat. no.: 732-6250)
- NAP-5 columns (Cytiva, Cat. no: 17-0853-01)
- Amicon ultra centrifugal filters (Merck Millipore, Burlington, MA, USA, Cat. no.: UFC9010)

### 2.3. Reagents Setup

Reagents, except for heat-sensitive liquids, should be sterilized by a combination of filtration and autoclaving.

Reagents, as well as equipment such as pipette tips and glassware, for the RNA preparation should be RNase-free.

Solutions for column chromatography are filter-sterilized. β-Me, DTT, PMSF, protease inhibitor cocktail, and others when indicated, are added to buffers immediately before use.

#### 2.3.1. eEF1A Preparation

**Lysis buffer:** 60 mM Tris-HCl (pH 7.5), 50 mM KCl, 5 mM MgCl_2_, 0.1 mM EDTA, 10% (vol/vol) glycerol, 20 μM GDP, 0.2 mM PMSF, and 2 mM DTT. Mix 60 mL of 1 M Tris-HCl (pH 7.5), 3.73 g of KCl, 5 mL of 1 M MgCl_2_, 200 μL of 0.5 M EDTA (pH 8.0), 100 mL of glycerol, 100 μL of 200 mM GDP, 2 mL of 100 mM PMSF, and 2 mL of 1 M DTT, and then add RNase-free water up to 1 L. Add 1 tablet of complete mini protease inhibitor cocktail (Roche) per 10 mL of buffer. Add GDP immediately before use.
**HiTrap SP A buffer:** 20 mM Tris-HCl (pH 7.5), 5 mM MgCl_2_, 25% (vol/vol) glycerol, 10 μM GDP, 0.1 mM PMSF, and 2 mM DTT. Mix 20 mL of 1 M Tris-HCl (pH 7.5), 5 mL of 1 M MgCl_2_, 250 mL of glycerol, 50 μL of 200 mM GDP, 1 mL of 100 mM PMSF, and 2 mL of 1 M DTT, and then, add RNase-free water up to 1 L. Add GDP immediately before use.
**HiTrap SP B buffer:** 20 mM Tris-HCl (pH 7.5), 1 M KCl, 5 mM MgCl_2_, 25% (vol/vol) glycerol, 10 μM GDP, 0.1 mM PMSF, and 2 mM DTT. Mix 20 mL of 1 M Tris-HCl (pH 7.5), 74.6 g of KCl, 5 mL of 1 M MgCl_2_, 250 mL of glycerol, 50 μL of 200 mM GDP, 1 mL of 100 mM PMSF, and 2 mL of 1 M DTT, and then, add RNase-free water up to 1 L. Add GDP immediately before use.
**HiTrap Butyl A buffer:** 20 mM HEPES-KOH (pH 7.5), 1.4 M (NH_4_)_2_SO_4_, 5 mM MgCl_2_, 10% (vol/vol) glycerol, 10 μM GDP, and 2 mM DTT. Mix 20 mL of 1 M HEPES-KOH (pH 7.5), 185 g of (NH_4_)_2_SO_4_, 5 mL of 1 M MgCl_2_, 100 mL of glycerol, 50 μL of 200 mM GDP, and 2 mL of 1 M DTT, and then, add RNase-free water up to 1 L. Add GDP immediately before use.
**HiTrap Butyl B buffer:** 20 mM HEPES-KOH (pH 7.5), 5 mM MgCl_2_, 10% (vol/vol) glycerol, 10 μM GDP, and 2 mM DTT. Mix 20 mL of 1 M HEPES-KOH (pH 7.5), 5 mL of 1 M MgCl_2_, 100 mL of glycerol, 50 μL of 200 mM GDP, and 2 mL of 1 M DTT, and then, add RNase-free water up to 1 L. Add GDP immediately before use.
**Stock buffer:** 20 mM HEPES-KOH (pH 7.5), 100 mM KCl, 5 mM MgCl_2_, 25% (vol/vol) glycerol, 10 μM GDP, 0.1 mM PMSF, and 2 mM DTT. Mix 20 mL of 1 M HEPES-KOH (pH 7.5), 7.46 g of KCl, 5 mL of 1 M MgCl_2_, 250 mL of glycerol, 50 μL of 200 mM GDP, 1 mL of 100 mM PMSF, and 2 mL of 1 M DTT, and then add RNase-free water up to 1 L. Add GDP immediately before use.

#### 2.3.2. eEF2 Preparation

**Lysis buffer:** 10 μM GDP, 0.2 mM PMSF, and 5 mM DTT in Y-PER Yeast Protein Extraction Reagent (Thermo Fisher Scientific). Mix 7.5 μL of 200 mM GDP, 300 μL of 100 mM PMSF, and 750 μL of 1 M DTT, and then, add Y-PER up to 150 mL. Add 1 tablet of complete mini protease inhibitor cocktail (Roche) per 10 mL of buffer.
**Ni-NTA wash Buffer I:** 50 mM HEPES-KOH (pH 7.5), 150 mM KCl, 20 mM Imidazole, 10% (vol/vol) glycerol, 10 μM GDP, 0.2 mM PMSF, and 3.5 mM β-Me. Mix 7.5 mL of 1 M HEPES-KOH (pH 7.5), 1.68 g of KCl, 1.2 mL of 2.5 M Imidazole (pH 7.5), 15 mL of glycerol, 7.5 μL of 200 mM GDP, 300 μL of 100 mM PMSF, and 38 μL of 14 M β-Me, and then, add RNase-free water up to 150 mL. Add GDP immediately before use.
**Ni-NTA wash Buffer II:** 50 mM HEPES-KOH (pH 7.5), 500 mM KCl, 20 mM Imidazole, 10% (vol/vol) glycerol, 10 μM GDP, 0.2 mM PMSF, and 3.5 mM β-Me. Mix 2.5 mL of 1 M HEPES-KOH (pH 7.5), 1.86 g of KCl, 400 μL of 2.5 M Imidazole (pH 7.5), 5 mL of glycerol, 2.5 μL of 200 mM GDP, 100 μL of 100 mM PMSF, and 12.5 μL of 14 M β-Me, and then, add RNase-free water up to 50 mL. Add GDP immediately before use.
**Ni-NTA elution buffer:** 50 mM HEPES-KOH (pH 7.5), 150 mM KCl, 250 mM Imidazole, 10% (vol/vol) glycerol, 10 μM GDP, 0.2 mM PMSF, and 3.5 mM β-Me. Mix 500 μL of 1 M HEPES-KOH (pH 7.5), 112 mg of KCl, 170 mg of solid imidazole, 1 mL of glycerol, 0.5 μL of 200 mM GDP, 20 μL of 100 mM PMSF, and 2.5 μL of 14 M β-Me, and then, add RNase-free water up to 10 mL. Add GDP immediately before use.
**Dialysis buffer:** 20 mM HEPES-KOH (pH 7.5), 100 mM KCl, 0.1 mM EDTA, 10% (vol/vol) glycerol, 10 μM GDP, 0.2 mM PMSF, and 1 mM DTT. Mix 20 mL of 1 M HEPES-KOH (pH 7.5), 7.46 g of KCl, 200 μL of 0.5 M EDTA (pH 8.0), 100 mL of glycerol, 50 μL of 200 mM GDP, 2 mL of 100 mM PMSF, and 1 mL of 1 M DTT, and then, add RNase-free water up to 1 L. Add GDP immediately before use.
**HiTrap Q A buffer:** 20 mM HEPES-KOH (pH 7.5), 0.1 mM EDTA, 10% (vol/vol) glycerol, 10 μM GDP, and 1 mM DTT. Mix 10 mL of 1 M HEPES-KOH (pH 7.5), 100 μL of 0.5 M EDTA (pH 8.0), 50 mL of glycerol, 25 μL of 200 mM GDP, and 500 μL of 1 M DTT, and then, add RNase-free water up to 500 mL. Add GDP immediately before use.
**HiTrap Q B buffer:** 20 mM HEPES-KOH (pH 7.5), 1 M KCl, 0.1 mM EDTA, 10% (vol/vol) glycerol, 10 μM GDP, and 1 mM DTT. Mix 10 mL of 1 M HEPES-KOH (pH 7.5), 37.3 g of KCl, 100 μL of 0.5 M EDTA (pH 8.0), 50 mL of glycerol, 25 μL of 200 mM GDP, and 500 μL of 1 M DTT, and then, add RNase-free water up to 500 mL. Add GDP immediately before use.
**Stock buffer:** 20 mM HEPES-KOH (pH 7.5), 100 mM KCl, 10% (vol/vol) glycerol, 10 μM GDP, and 1 mM DTT. Mix 20 mL of 1 M HEPES-KOH (pH 7.5), 7.46 g of KCl, 100 mL of glycerol, 50 μL of 200 mM GDP, and 1 mL of 1 M DTT, and then, add RNase-free water up to 1 L. Add GDP immediately before use.

#### 2.3.3. eEF3 Preparation

**Lysis buffer:** 50 mM HEPES-KOH (pH 7.5), 500 mM KCl, 10% (vol/vol) glycerol, 0.2 mM PMSF, and 7 mM β-Me. Mix 50 mL of 1 M HEPES-KOH (pH 7.5), 37.3 g of KCl, 100 mL of glycerol, 2 mL of 100 mM PMSF, and 500 μL of 14 M β-Me, and then, add RNase-free water up to 1 L. Add 1 tablet of complete mini protease inhibitor cocktail (Roche) per 50 mL of buffer.
**Ni-NTA wash buffer:** 50 mM HEPES-KOH (pH 7.5), 1 M KCl, 20 mM Imidazole, 10% (vol/vol) glycerol, 0.2 mM PMSF, and 3.5 mM β-Me. Mix 17.5 mL of 1 M HEPES-KOH (pH 7.5), 26.1 g of KCl, 2.8 mL of 2.5 M Imidazole (pH 7.5), 35 mL of glycerol, 700 μL of 100 mM PMSF, and 87.5 μL of 14 M β-Me, and then, add RNase-free water up to 350 mL.
**Ni-NTA elution buffer:** 50 mM HEPES-KOH (pH 7.5), 100 mM KCl, 250 mM Imidazole, 10% (vol/vol) glycerol, 0.2 mM PMSF, and 3.5 mM β-Me. Mix 1 mL of 1 M HEPES-KOH (pH 7.5), 149 mg of KCl, 340 mg of solid Imidazole, 2 mL of glycerol, 40 μL of 100 mM PMSF, and 5 μL of 14 M β-Me, and then, add RNase-free water up to 20 mL.
**Dialysis buffer:** 20 mM HEPES-KOH (pH 7.5), 150 mM KCl, 0.1 mM EDTA, 10% (vol/vol) glycerol, and 7 mM β-Me. Mix 20 mL of 1 M HEPES-KOH (pH 7.5), 11.2 g of KCl, 200 μL of 0.5 M EDTA (pH 8.0), 100 mL of glycerol, and 500 μL of 14 M β-Me, and then, add RNase-free water up to 1 L.
**HiTrap Q A buffer:** 20 mM HEPES-KOH (pH 7.5), 10% (vol/vol) glycerol, and 5 mM β-Me. Mix 10 mL of 1 M HEPES-KOH (pH 7.5), 50 mL of glycerol, and 179 μL of 14 M β-Me, and then, add RNase-free water up to 500 mL.
**HiTrap Q B buffer:** 20 mM HEPES-KOH (pH 7.5), 1 M KCl, 10% (vol/vol) glycerol, and 5 mM β-Me. Mix 10 mL of 1 M HEPES-KOH (pH 7.5), 37.3 g of KCl, 50 mL of glycerol, and 179 μL of 14 M β-Me, and then, add RNase-free water up to 500 mL.
**Stock buffer:** 20 mM HEPES-KOH (pH 7.5), 100 mM KCl, 10% (vol/vol) glycerol, and 3.5 mM β-Me. Mix 20 mL of 1 M HEPES-KOH (pH 7.5), 7.46 g of KCl, 100 mL of glycerol, and 250 μL of 14 M β-Me, and then, add RNase-free water up to 1 L.

#### 2.3.4. eRF1 Preparation

**Lysis buffer:** 50 mM Tris-HCl (pH 7.5), 100 mM KCl, 10% (vol/vol) glycerol, 0.1 mM PMSF, and 3.5 mM β-Me. Mix 50 mL of 1 M Tris-HCl (pH 7.5), 7.46 g of KCl, 100 mL of glycerol, 1 mL of 100 mM PMSF, and 250 μL of 14 M β-Me, and then, add RNase-free water up to 1 L. Add 1 tablet of complete mini protease inhibitor cocktail (Roche) per 50 mL of buffer.
**Ni-NTA wash buffer:** 50 mM Tris-HCl (pH 7.5), 1 M NH_4_Cl, 10 mM Imidazole, 10% (vol/vol) glycerol, and 3.5 mM β-Me. Mix 25 mL of 1 M Tris-HCl (pH 7.5), 26.7 g of NH_4_Cl, 2 mL of 2.5 M Imidazole (pH 7.5), 50 mL of glycerol, and 125 μL of 14 M β-Me, and then, add RNase-free water up to 500 mL.
**Ni-NTA elution buffer:** 50 mM Tris-HCl (pH 7.5), 100 mM KCl, 200 mM Imidazole, 10% (vol/vol) glycerol, and 3.5 mM β-Me. Mix 1.5 mL of 1 M Tris-HCl (pH 7.5), 224 mg of KCl, 408 mg of solid imidazole, 3 mL of glycerol, and 7.5 μL of 14 M β-Me, and then, add RNase-free water up to 30 mL.
**HiTrap Q A buffer:** 20 mM HEPES-KOH (pH 7.5), 10% (vol/vol) glycerol, and 7 mM β-Me. Mix 20 mL of 1 M HEPES-KOH (pH 7.5), 100 mL of glycerol, and 500 μL of 14 M β-Me, and then, add RNase-free water up to 1 L.
**HiTrap Q B buffer:** 20 mM HEPES-KOH (pH 7.5), 1 M KCl, 10% (vol/vol) glycerol, and 7 mM β-Me. Mix 20 mL of 1 M HEPES-KOH (pH 7.5), 74.6 g of KCl, 100 mL of glycerol, and 500 μL of 14 M β-Me, and then, add RNase-free water up to 1 L.
**Stock buffer:** 20 mM HEPES-KOH (pH 7.5), 100 mM KCl, 10% (vol/vol) glycerol, and 7 mM β-Me. Mix 20 mL of 1 M HEPES-KOH (pH 7.5), 7.46 g of KCl, 100 mL of glycerol, and 500 μL of 14 M β-Me, and then, add RNase-free water up to 1 L.

#### 2.3.5. eRF3∆165 Preparation

**Lysis buffer:** 50 mM Tris-HCl (pH 7.5), 100 mM KCl, 7 mM MgCl_2_, 10 mM imidazole, 10% (vol/vol) glycerol, 50 μM GDP, 0.1 mM PMSF, and 3.5 mM β-Me. Mix 50 mL of 1 M Tris-HCl (pH 7.5), 7.46 g of KCl, 7 mL of 1 M MgCl_2_, 4 mL of 2.5 M imidazole (pH 7.5), 100 mL of glycerol, 250 μL of 200 mM GDP, 1 mL of 100 mM PMSF, and 250 μL of 14 M β-Me, and then, add RNase-free water up to 1 L. Add 1 tablet of complete mini protease inhibitor cocktail (Roche) per 50 mL of buffer. Add GDP immediately before use.
**Ni-NTA wash buffer:** 50 mM Tris-HCl (pH 7.5), 1 M NH_4_Cl, 7 mM MgCl_2_, 10 mM imidazole, 10% (vol/vol) glycerol, 50 μM GDP, and 3.5 mM β-Me. Mix 25 mL of 1 M Tris-HCl (pH 7.5), 26.7 g of NH_4_Cl, 3.5 mL of 1 M MgCl_2_, 2 mL of 2.5 M imidazole (pH 7.5), 50 mL of glycerol, 125 μL of 200 mM GDP, and 125 μL of 14 M β-Me, and then, add RNase-free water up to 500 mL. Add GDP immediately before use.
**Ni-NTA elution buffer:** 50 mM Tris-HCl (pH 7.5), 100 mM KCl, 7 mM MgCl_2_, 150 mM imidazole, 10% (vol/vol) glycerol, 50 μM GDP, and 3.5 mM β-Me. Mix 1.5 mL of 1 M Tris-HCl (pH 7.5), 224 mg of KCl, 210 μL of 1 M MgCl_2_, 306 mg of solid imidazole, 3 mL of glycerol, 7.5 μL of 200 mM GDP, and 7.5 μL of 14 M β-Me, and then, add RNase-free water up to 30 mL. Add GDP immediately before use.
**HiTrap Q A buffer:** 20 mM HEPES-KOH (pH 7.5), 1 mM MgCl_2_, 0.1 mM EDTA, 10% (vol/vol) glycerol, 50 μM GDP and 7 mM β-Me. Mix 20 mL of 1 M HEPES-KOH (pH 7.5), 1 mL of 1 M MgCl_2_, 200 μL of 0.5 M EDTA (pH 8.0), 100 mL of glycerol, 250 μL of 200 mM GDP, and 500 μL of 14 M β-Me, and then, add RNase-free water up to 1 L. Add GDP immediately before use.
**HiTrap Q B buffer:** 20 mM HEPES-KOH (pH 7.5), 1 M KCl, 1 mM MgCl_2_, 0.1 mM EDTA, 10% (vol/vol) glycerol, 50 μM GDP and 7 mM β-Me. Mix 20 mL of 1 M HEPES-KOH (pH 7.5), 74.6 g of KCl, 1 mL of 1 M MgCl_2_, 200 μL of 0.5 M EDTA (pH 8.0), 100 mL of glycerol, 250 μL of 200 mM GDP, and 500 μL of 14 M β-Me, and then, add RNase-free water up to 1 L. Add GDP immediately before use.
**Stock buffer:** 20 mM HEPES-KOH (pH 7.5), 100 mM KCl, 1 mM MgCl_2_, 0.1 mM EDTA, 10% (vol/vol) glycerol, 50 μM GDP and 7 mM β-Me. Mix 20 mL of 1 M HEPES-KOH (pH 7.5), 7.46 g of KCl, 1 mL of 1 M MgCl_2_, 200 μL of 0.5 M EDTA (pH 8.0), 100 mL of glycerol, 250 μL of 200 mM GDP, and 500 μL of 14 M β-Me, and then, add RNase-free water up to 1 L. Add GDP immediately before use.

#### 2.3.6. Dom34 Preparation

**Lysis buffer:** 50 mM Tris-HCl (pH 7.5), 100 mM KCl, 10% (vol/vol) glycerol, 0.1 mM PMSF, and 3.5 mM β-Me. Mix 50 mL of 1 M Tris-HCl (pH 7.5), 7.46 g of KCl, 100 mL of glycerol, 1 mL of 100 mM PMSF, and 250 μL of 14 M β-Me, and then, add RNase-free water up to 1 L. Add 1 tablet of complete mini protease inhibitor cocktail (Roche) per 50 mL of buffer.
**Ni-NTA wash buffer:** 50 mM Tris-HCl (pH 7.5), 1 M NH_4_Cl, 10 mM Imidazole, 10% (vol/vol) glycerol, and 3.5 mM β-Me. Mix 25 mL of 1 M Tris-HCl (pH 7.5), 26.7 g of NH_4_Cl, 2 mL of 2.5 M Imidazole (pH 7.5), 50 mL of glycerol, and 125 μL of 14 M β-Me, and then, add RNase-free water up to 500 mL.
**Ni-NTA elution buffer:** 50 mM Tris-HCl (pH 7.5), 100 mM KCl, 200 mM Imidazole, 10% (vol/vol) glycerol, and 3.5 mM β-Me. Mix 1.5 mL of 1 M Tris-HCl (pH 7.5), 224 mg of KCl, 408 mg of solid imidazole, 3 mL of glycerol, and 7.5 μL of 14 M β-Me, and then, add RNase-free water up to 30 mL.
**HiTrap Q A buffer:** 20 mM HEPES-KOH (pH 7.5), 10% (vol/vol) glycerol, and 7 mM β-Me. Mix 20 mL of 1 M HEPES-KOH (pH 7.5), 100 mL of glycerol, and 500 μL of 14 M β-Me, and then, add RNase-free water up to 1 L.
**HiTrap Q B buffer:** 20 mM HEPES-KOH (pH 7.5), 1 M KCl, 10% (vol/vol) glycerol, and 7 mM β-Me. Mix 20 mL of 1 M HEPES-KOH (pH 7.5), 74.6 g of KCl, 100 mL of glycerol, and 500 μL of 14 M β-Me, and then, add RNase-free water up to 1 L.
**Stock buffer:** 20 mM HEPES-KOH (pH 7.5), 200 mM KCl, 10% (vol/vol) glycerol, and 7 mM β-Me. Mix 20 mL of 1 M HEPES-KOH (pH 7.5), 14.9 g of KCl, 100 mL of glycerol, and 500 μL of 14 M β-Me, and then, add RNase-free water up to 1 L.

#### 2.3.7. Hbs1 Preparation

**Lysis buffer:** 50 mM Tris-HCl (pH 7.5), 100 mM KCl, 7 mM MgCl_2_, 10 mM imidazole, 10% (vol/vol) glycerol, 50 μM GDP, 0.1 mM PMSF, and 3.5 mM β-Me. Mix 50 mL of 1 M Tris-HCl (pH 7.5), 7.46 g of KCl, 7 mL of 1 M MgCl_2_, 4 mL of 2.5 M imidazole (pH 7.5), 100 mL of glycerol, 250 μL of 200 mM GDP, 1 mL of 100 mM PMSF, and 250 μL of 14 M β-Me, and then, add RNase-free water up to 1 L. Add 1 tablet of complete mini protease inhibitor cocktail (Roche) per 50 mL of buffer. Add GDP immediately before use.
**Ni-NTA wash buffer:** 50 mM Tris-HCl (pH 7.5), 1 M NH_4_Cl, 7 mM MgCl_2_, 10 mM imidazole, 10% (vol/vol) glycerol, 50 μM GDP, and 3.5 mM β-Me. Mix 15 mL of 1 M Tris-HCl (pH 7.5), 16 g of NH_4_Cl, 2.1 mL of 1 M MgCl_2_, 1.2 mL of 2.5 M imidazole (pH 7.5), 30 mL of glycerol, 75 μL of 200 mM GDP, and 75 μL of 14 M β-Me, and then, add RNase-free water up to 300 mL. Add GDP immediately before use.
**Ni-NTA elution buffer:** 50 mM Tris-HCl (pH 7.5), 100 mM KCl, 7 mM MgCl_2_, 150 mM imidazole, 10% (vol/vol) glycerol, 50 μM GDP, and 3.5 mM β-Me. Mix 1 mL of 1 M Tris-HCl (pH 7.5), 149 mg of KCl, 140 μL of 1 M MgCl_2_, 204 mg of solid imidazole, 2 mL of glycerol, 5 μL of 200 mM GDP, and 5 μL of 14 M β-Me, and then, add RNase-free water up to 20 mL. Add GDP immediately before use.
**Mono Q A buffer:** 20 mM HEPES-KOH (pH 7.5), 1 mM MgCl_2_, 0.1 mM EDTA, 10% (vol/vol) glycerol, 50 μM GDP and 7 mM β-Me. Mix 20 mL of 1 M HEPES-KOH (pH 7.5), 1 mL of 1 M MgCl_2_, 200 μL of 0.5 M EDTA (pH 8.0), 100 mL of glycerol, 250 μL of 200 mM GDP, and 500 μL of 14 M β-Me, and then, add RNase-free water up to 1 L. Add GDP immediately before use.
**HiTrap Q B buffer:** 20 mM HEPES-KOH (pH 7.5), 1 M KCl, 1 mM MgCl_2_, 0.1 mM EDTA, 10% (vol/vol) glycerol, 50 μM GDP and 7 mM β-Me. Mix 20 mL of 1 M HEPES-KOH (pH 7.5), 74.6 g of KCl, 1 mL of 1 M MgCl_2_, 200 μL of 0.5 M EDTA (pH 8.0), 100 mL of glycerol, 250 μL of 200 mM GDP, and 500 μL of 14 M β-Me, and then, add RNase-free water up to 1 L. Add GDP immediately before use.
**Stock buffer:** 20 mM HEPES-KOH (pH 7.5), 100 mM KCl, 1 mM MgCl_2_, 0.1 mM EDTA, 10% (vol/vol) glycerol, 50 μM GDP and 7 mM β-Me. Mix 20 mL of 1 M HEPES-KOH (pH 7.5), 7.46 g of KCl, 1 mL of 1 M MgCl_2_, 200 μL of 0.5 M EDTA (pH 8.0), 100 mL of glycerol, 250 μL of 200 mM GDP, and 500 μL of 14 M β-Me, and then, add RNase-free water up to 1 L. Add GDP immediately before use.

#### 2.3.8. Rli1 Preparation

**Lysis buffer:** 75 mM HEPES-KOH (pH 7.5), 1 M NH_4_Cl, 20 mM imidazole, 20% (vol/vol) glycerol, 0.1 mM PMSF, and 5 mM β-Me. Mix 75 mL of 1 M HEPES-KOH (pH 7.5), 53.5 g of NH_4_Cl, 8 mL of 2.5 M imidazole (pH 7.5), 200 mL of glycerol, 1 mL of 100 mM PMSF, and 357 μL of 14 M β-Me, and then, add RNase-free water up to 1 L. Add 1 tablet of complete mini protease inhibitor cocktail (Roche) per 10 mL of buffer.
**Ni-NTA wash buffer:** 50 mM HEPES-KOH (pH 7.5), 1 M NH_4_Cl, 20 mM imidazole, 20% (vol/vol) glycerol, and 5 mM β-Me. Mix 50 mL of 1 M HEPES-KOH (pH 7.5), 53.5 g of NH_4_Cl, 8 mL of 2.5 M imidazole (pH 7.5), 200 mL of glycerol, and 357 μL of 14 M β-Me, and then, add RNase-free water up to 1 L.
**Ni-NTA wash buffer + 1% (vol/vol) TWEEN 20:** Add 5 mL of TWEEN 20 to 500 mL of Ni-NTA wash buffer.
**Ni-NTA elution buffer:** 50 mM HEPES-KOH (pH 7.5), 1 M NH_4_Cl, 300 mM imidazole, 20% (vol/vol) glycerol, and 5 mM β-Me. Mix 2.5 mL of 1 M HEPES-KOH (pH 7.5), 2.67 g of NH_4_Cl, 1.02 g of solid imidazole, 10 mL of glycerol, and 17.9 μL of 14 M β-Me, and then, add RNase-free water up to 50 mL.
**HiTrap SP A buffer:** 20 mM MES-KOH (pH 6.6), 100 mM KCl, 0.1 mM EDTA, 25% (vol/vol) glycerol, and 7 mM β-Me. Mix 10 mL of 1 M MES-KOH (pH 6.6), 3.73 g of KCl, 100 μL of 0.5 M EDTA (pH 8.0), 125 mL of glycerol, and 250 μL of 14 M β-Me, and then, add RNase-free water up to 500 mL.
**HiTrap SP B buffer:** 20 mM MES-KOH (pH 6.6), 1 M KCl, 0.1 mM EDTA, 25% (vol/vol) glycerol, and 7 mM β-Me. Mix 10 mL of 1 M MES-KOH (pH 6.6), 37.3 g of KCl, 100 μL of 0.5 M EDTA (pH 8.0), 125 mL of glycerol, and 250 μL of 14 M β-Me, and then, add RNase-free water up to 500 mL.
**Stock buffer:** 20 mM HEPES-KOH (pH 7.5), 200 mM KCl, 0.1 mM EDTA, 25% (vol/vol) glycerol, 0.1 mM PMSF, and 7 mM β-Me. Mix 20 mL of 1 M HEPES-KOH (pH 7.5), 14.9 g of KCl, 200 μL of 0.5 M EDTA (pH 8.0), 250 mL of glycerol, 1 mL of 100 mM PMSF, and 500 μL of 14 M β-Me, and then, add RNase-free water up to 1 L.

#### 2.3.9. Ribosome Preparation

**Lysis buffer:** 20 mM HEPES-KOH (pH 7.5), 100 mM KOAc (pH 7.5), 2.5 mM Mg(OAc)_2_, 0.5 mg/mL heparin, and 2 mM DTT. Mix 20 mL of 1 M HEPES-KOH (pH 7.5), 33.3 mL of 3 M KOAc (pH 7.5), 2.5 mL of 1 M Mg(OAc)_2_, 5 mL of 100 mg/mL heparin, and 2 mL of 1 M DTT, and then, add RNase-free water up to 1 L. Add 1 tablet of complete mini protease inhibitor cocktail (Roche) per 10 mL of buffer. The requisite amount of the buffer is approximately 1.2 L. Add heparin immediately before use.
**Sucrose cushion Buffer I:** 20 mM HEPES-KOH (pH 7.5), 100 mM KCl, 20 mM MgCl_2_, 1 M sucrose, 0.5 mg/mL heparin, 0.1 mM PMSF, and 2 mM DTT. Mix 500 μL of 1 M HEPES-KOH (pH 7.5), 186 mg of KCl, 500 μL of 1 M MgCl_2_, 8.56 g of sucrose, 125 μL of 100 mg/mL heparin, 25 μL of 100 mM PMSF, and 50 μL of 1 M DTT, and then, add RNase-free water up to 25 mL. Add heparin immediately before use.
>10 mM puromycin: Dissolve 54.4 mg of puromycin∙2HCl in 10 mL Puromycin treatment buffer. Store in approximately single-use aliquots (310 μL) at −80 °C.
**Puromycin treatment buffer:** 20 mM HEPES-KOH (pH 7.5), 50 mM (NH_4_)_2_SO_4_, 20 mM MgCl_2_, and 2 mM DTT. Mix 200 μL of 1 M HEPES-KOH (pH 7.5), 66.1 mg of solid (NH_4_)_2_SO_4_, 200 μL of 1 M MgCl_2_, and 20 μL of 1 M DTT, and then, add RNase-free water up to 10 mL.
**HiTrap Butyl A buffer:** 20 mM HEPES-KOH (pH 7.5), 1.4 M (NH_4_)_2_SO_4_, 50 mM MgCl_2_, and 2 mM DTT. Mix 20 mL of 1 M HEPES-KOH (pH 7.5), 185 g of (NH_4_)_2_SO_4_, 50 mL of 1 M MgCl_2_, and 2 mL of 1 M DTT, and then, add RNase-free water up to 1 L.
**HiTrap Butyl B buffer:** 20 mM HEPES-KOH (pH 7.5), 20 mM MgCl_2_, and 2 mM DTT. Mix 20 mL of 1 M HEPES-KOH (pH 7.5), 20 mL of 1 M MgCl_2_, and 2 mL of 1 M DTT, and then, add RNase-free water up to 1 L.
**Sucrose cushion Buffer II:** 20 mM HEPES-KOH (pH 7.5), 100 mM KCl, 10 mM MgCl_2_, 1 M sucrose, and 2 mM DTT. Mix 1 mL of 1 M HEPES-KOH (pH 7.5), 373 mg of KCl, 500 μL of 1 M MgCl_2_, 17.1 g of sucrose, and 100 μL of 1 M DTT, and then, add RNase-free water up to 50 mL.
**5/100 buffer:** 20 mM HEPES-KOH (pH 7.5), 100 mM KCl, 5 mM MgCl_2_, 0.25 mM EDTA, and 2 mM DTT. Mix 200 μL of 1 M HEPES-KOH (pH 7.5), 74.6 mg of KCl, 50 μL of 1 M MgCl_2_, 5 μL of 0.5 M EDTA (pH 8.0), and 20 μL of 1 M DTT, and then, add RNase-free water up to 10 mL.

#### 2.3.10. tRNA Preparation

**Lysis buffer:** 20 mM HEPES-KOH (pH 7.5), 100 mM KOAc (pH 7.5), and 2 mM Mg(OAc)_2_. Mix 20 mL of 1 M HEPES-KOH (pH 7.5), 33.3 mL of 3 M KOAc (pH 7.5), and 2 mL of 1 M Mg(OAc)_2_, and then, add RNase-free water up to 1 L.
**1 mM Tris-HCl (pH 7.5)–10 mM Mg(OAc)_2_:** Mix 50 μL of 1 M Tris-HCl (pH 7.5) and 500 μL of 1 M Mg(OAc)_2_, and then, add RNase-free water up to 50 mL.
**Phenol saturated with 1 mM Tris-HCl (pH 7.5)–10 mM Mg(OAc)_2_:** Heat solid phenol at 50–60 °C until phenol dissolves completely. (i) Add an equal amount of 1 M Tris-HCl (pH 7.5) to the phenol solution, mixing rigorously with a magnetic stirrer for about 1 h at room temperature. (ii) Remove the aqueous (upper) phase and measure the pH of the aqueous phase. Repeat steps (i) and (ii) until the pH reaches 7.5. (iii) Add an equal amount of 1 mM Tris-HCl (pH 7.5)–10 mM Mg(OAc)_2_ to the phenol solution, mixing rigorously with a magnetic stirrer for about 1 h at room temperature. (iv) Remove the aqueous (upper) phase and measure the pH of the aqueous phase. Repeat steps (iii) and (iv) twice, and check that the pH of the aqueous phase is 7.5. Remove the aqueous phase, leaving a small amount on top of the phenol. Store away from light at 4 °C, where it usually remains stable for months.
**HiTrap Q A buffer:** 20 mM Tris-HCl (pH 7.5), 200 mM NaCl, 8 mM MgCl_2_, and 0.1 mM EDTA. Mix 20 mL of 1 M Tris-HCl (pH 7.5), 11.7 g of NaCl, 8 mL of 1 M MgCl_2_, and 200 μL of 0.5 M EDTA (pH 8.0), and then, add RNase-free water up to 1 L.
**HiTrap Q B buffer:** 20 mM Tris-HCl (pH 7.5), 1 M NaCl, 8 mM MgCl_2_, and 0.1 mM EDTA. Mix 20 mL of 1 M Tris-HCl (pH 7.5), 58.4 g of NaCl, 8 mL of 1 M MgCl_2_, and 200 μL of 0.5 M EDTA (pH 8.0), and then, add RNase-free water up to 1 L.

#### 2.3.11. Preparation of Aminoacyl-tRNAs

**Lysis buffer (for S100 preparation):** 60 mM Tris-OAc (pH 7.0), 50 mM NH_4_Cl, 5 mM Mg(OAc)_2_, 0.1 mM EDTA, 10% (vol/vol) glycerol, 2 mM spermidine, 0.1 mM PMSF, and 10 mM β-Me. Mix 60 mL of 1 M Tris-OAc (pH 7.0), 2.7 g of NH_4_Cl, 5 mL of 1 M Mg(OAc)_2_, 200 μL of 0.5 M EDTA (pH 8.0), 100 mL of glycerol, 2 mL of 1 M spermidine, 1 mL of 100 mM PMSF, and 714 μL of 14 M β-Me, and then, add RNase-free water up to 1 L. Add 1 tablet of complete mini protease inhibitor cocktail (Roche) per 10 mL of buffer. The requisite amount of the buffer is approximately 1.2 L. Add spermidine immediately before use.
**Buffer A (for S100 preparation):** 20 mM HEPES-KOH (pH 8.0), 5 mM NaCl, 5 mM MgCl_2_, 0.1 mM EDTA, 10% (vol/vol) glycerol, 0.1 mM PMSF, and 1 mM DTT. Mix 20 mL of 1 M HEPES-KOH (pH 8.0), 292 mg of NaCl, 5 mL of 1 M MgCl_2_, 200 μL of 0.5 M EDTA (pH 8.0), 100 mL of glycerol, 1 mL of 100 mM PMSF, and 1 mL of 1 M DTT, and then, add RNase-free water up to 1 L. Add 1 tablet of complete mini protease inhibitor cocktail (Roche) per 10 mL of buffer. The requisite amount of the buffer is approximately 4 L.
**Buffer A (250 mM NaCl) (for S100 preparation):** 20 mM HEPES-KOH (pH 8.0), 250 mM NaCl, 5 mM MgCl_2_, 0.1 mM EDTA, 10% (vol/vol) glycerol, 0.1 mM PMSF, and 1 mM DTT. Mix 2 mL of 1 M HEPES-KOH (pH 8.0), 1.46 g of NaCl, 500 μL of 1 M MgCl_2_, 20 μL of 0.5 M EDTA (pH 8.0), 10 mL of glycerol, 100 μL of 100 mM PMSF, and 100 μL of 1 M DTT, and then, add RNase-free water up to 100 mL. Add 1 tablet of complete mini protease inhibitor cocktail (Roche) per 10 mL of buffer.
**Solution D:** 4 M guanidine thiocyanate, 25 mM sodium citrate (pH 7.0), 0.5% (wt/vol) sarcosyl, and 85 mM β-Me. Mix 1.42 g of guanidine thiocyanate, 22.1 mg of trisodium citrate dihydrate, 15 mg of N-Lauroylsarcosine sodium salt, and 18.2 μL of 14 M β-Me, and then, add RNase-free water up to 3 mL. Prepare fresh before use.
**Phenol saturated with 50 mM KOAc (pH 5.5):** Heat solid phenol at 50–60 °C until phenol dissolves completely. (i) Add an equal amount of 50 mM KOAc (pH 5.5) to the phenol solution, mixing rigorously with a magnetic stirrer for about 1 h at room temperature. (ii) Remove the aqueous (upper) phase and measure the pH of the aqueous phase. Repeat Steps (i) and (ii) until the pH reaches 5.5. Remove the aqueous phase, leaving a small amount on top of the phenol. Store away from light at 4 °C, where it usually remains stable for months.
**CIA (Chloroform:Isoamyl alcohol, 49:1):** Mix 980 μL of chloroform and 20 μL of isoamyl alcohol.
**0.3 M KOAc (pH 5.5)—5% (vol/vol) isopropanol:** Mix 2.5 mL of 3 M KOAc (pH 5.5) and 1.25 mL of isopropanol, and then, add RNase-free water up to 25 mL. Prepare fresh before use.

#### 2.3.12. In Vitro Translation

**1.5 mM 18 a.a. (1.5 mM each 18 amino acids without Cys and Met):** Mix 1.34 mg of Ala, 2.61 mg of Arg, 2.25 mg of Asn, 2.00 mg of Asp, 2.19 mg of Gln, 2.21 mg of Glu, 1.13 mg of Gly, 2.33 mg of His, 1.97 mg of Ile, 1.97 mg of Leu, 2.19 mg of Lys, 2.48 mg of Phe, 1.73 mg of Pro, 1.58 mg of Ser, 1.79 mg of Thr, 3.06 mg of Trp, 2.72 mg of Tyr, and 1.76 mg of Val, and then, add 0.1 M HEPES-KOH (pH 7.5) up to 10 mL. Sterilize the solution with filtration. Store in approximately single-use aliquots (20 μL) at −80 °C.
**10 mM Cys:** To 12.1 mg of Cys, add RNase-free water up to 10 mL. Sterilize the solution with filtration. Store in approximately single-use aliquots (20 μL) at −80 °C.
**10x NTPs:** Mix 200 μL of 100 mM ATP, 200 μL of 100 mM GTP, 100 μL of 100 mM CTP, 100 μL of 100 mM UTP, and 400 μL of RNase-free water. Sterilize the solution with filtration. Store in approximately single-use aliquots (20 μL) at −80 °C.
**20 mM ATP, GTP:** Mix 200 μL of 100 mM ATP, 200 μL of 100 mM GTP, and 600 μL of RNase-free water. Sterilize the solution with filtration. Store in approximately single-use aliquots (20 μL) at −80 °C.
**12% PURE buffer (without Mg and SPD):** Mix 1.5 mL of 1 M HEPES-KOH (pH 7.5), 1.5 mL of 2 M L-Glutamic acid potassium salt monohydrate, 30 μL of 1 M DTT, and 570 μL of RNase-free water. Sterilize the solution with filtration. Store in approximately single-use aliquots (20 μL) at −80 °C.
**2 M L-Glutamic acid potassium salt monohydrate:** To 4.06 mg of L-Glutamic acid potassium salt monohydrate, add RNase-free water up to 10 mL. Sterilize the solution with filtration. Store at −80 °C.
**1 M Creatine Phosphate:** To 3.27 g of creatine phosphate, add RNase-free water up to 10 mL. Sterilize the solution with filtration. Store in approximately single-use aliquots (20 μL) at −80 °C.
**250 mM Mg(OAc)_2_:** To 536 mg of magnesium acetate tetrahydrate, add RNase-free water up to 10 mL. Sterilize the solution with filtration. Store in approximately single-use aliquots (20 μL) at −80 °C.
**1 M Spermidine:** To 1.45 g of spermidine, add RNase-free water up to 10 mL. Store at −80 °C.
**20 mM Spermidine**: Mix 200 μL of 1 M spermidine and 9.8 mL of RNase-free water. Store in approximately single-use aliquots (20 μL) at −80 °C.
**1 M Spermine:** To 2.02 g of spermine, add RNase-free water up to 10 mL. Store at −80 °C.
**10 mM Spermine**: Mix 100 μL of 1 M spermine and 9.9 mL of RNase-free water. Store in approximately single-use aliquots (20 μL) at −80 °C.
**1 mM Met:** To 1.49 mg of Met, add RNase-free water up to 10 mL. Sterilize the solution with filtration. Store in approximately single-use aliquots (20 μL) at −80 °C.
**5 mg/mL RNase A:** To 7.5 mg of RNaseA, add buffer (20 mM HEPES-KOH (pH 7.5), 100 mM KOAc (pH 7.5), 2 mM Mg(OAc)_2_) up to 1.5 mL. Store in aliquots (50 μL) at −30 °C.
**0.1 mg/mL RNase A:** Mix 200 μL of 5 mg/mL RNase A and 9.8 mL of buffer (20 mM HEPES-KOH (pH 7.5), 100 mM KOAc (pH 7.5), 2 mM Mg(OAc)_2_). Store in aliquots (1 mL) at −30 °C.

#### 2.3.13. Cell Culture

**LB medium:** Mix 10 g of bacto tryptone, 5 g of bacto yeast extract, and 10 g of NaCl, and then, add water up to 1 L. Thereafter, autoclave it.
**2xYT medium:** Mix 16 g of bacto tryptone, 10 g of bacto yeast extract, and 5 g of NaCl, and then, add water up to 1 L. Thereafter, autoclave it.
**YPD medium:** Mix 9 g of bacto yeast extract, 18 g of hipolypepton, and 18 g of D(+)-glucose, and then, add water up to 900 mL. Thereafter, autoclave it. Bacto peptone (Thermo Fisher Scientific) can be used instead of hipolypepton (Wako).
**SC-Ura medium containing 1% (wt/vol) raffinose:** To prepare 400 mL of medium, mix 2.67 g of difco yeast nitrogen base without amino acids and 0.77 g of dropout mix without Ura, and then, add water up to 350 mL. Add 1 M NaOH (approximately 4 mL) into the medium, until the pH reaches 7–8. After pH adjustment, add water up to 360 mL into the medium. Thereafter, autoclave it. Cool the medium to room temperature and add 40 mL of 10% (wt/vol) raffinose. To prepare 720 mL of medium, mix 5.33 g of difco yeast nitrogen base without amino acids and 1.54 g of dropout mix without Ura, and then, add water up to 600 mL. Add 1 M NaOH (approximately 7 mL) into the medium, until the pH reaches 7–8. After pH adjustment, add water up to 640 mL into the medium. Thereafter, autoclave it. Cool the medium to room temperature and add 80 mL of 10% (wt/vol) raffinose.
**10% (wt/vol) raffinose:** To 100 g of D(+)-raffinose, add sterile water up to 1 L. Sterilize the solution by filtration.
**20% (wt/vol) galactose:** To 200 g of D(+)-galactose, add sterile water up to 1 L. Sterilize the solution by filtration.
**Dropout mix without Ura:** Mix 2 g of Adenine, 8 g of L-Inositol, 8 g of Ala, 8 g of Arg, 8 g of Asn, 8 g of Asp, 8 g of Cys, 8 g of Gln, 8 g of Glu, 8 g of Gly, 8 g of His, 8 g of Ile, 40 g of Leu, 8 g of Lys, 8 g of Met, 8 g of Phe, 8 g of Pro, 8 g of Ser, 8 g of Thr, 8 g of Trp, 8 g of Tyr, 8 g of Val, and 0.8 g of p-Aminobenzoic acid. Grind the mixture until a fine powder is formed. Store away from light at room temperature, where it usually remains stable for months.

#### 2.3.14. Others

**2x RNA loading dye:** 0.05% (wt/vol) bromophenol blue (BPB), 0.05% (wt/vol) Xylene cyanole FF (XC), 0.5 mM EDTA, and 7 M urea. Mix 5 mg of BPB, 5 mg of XC, 10 μL of 0.5 M EDTA (pH 8.0), and 4.2 g of urea, and then, add RNase-free water up to 10 mL. Can be stored at −30 °C.
**5x LiDS dye:** 5% (wt/vol) lithium dodecyl sulfate (LiDS), 25% (vol/vol) glycerol, 100 mM Tris-HCl (pH 6.8), 0.025% (wt/vol) BPB, and 875 mM β-Me. Mix 500 mg of LiDS, 2.5 mL of glycerol, 1 mL of 1 M Tris-HCl (pH 6.8), 2.5 mg of BPB, and 625 μL of 14 M β-Me, and then, add RNase-free water up to 10 mL. Can be stored at −30 °C.

## 3. Procedure

### 3.1. Preparation of Yeast Translation Factors

All translation factors are checked for purity by SDS-PAGE analysis after purification (see [18] for the typical results). The concentrations of purified translation factors are determined by the Bradford Assay or by measuring the absorption spectrum and using the absorption coefficient (Table S1). The yields of the factors are summarized in Table S2. Verify the activities of the purified factors, according to Section 3.4.3, for the translation reaction method, and refer to Table S4 for the appropriate translation reaction condition for each translation factor.

#### 3.1.1. eEF1A

**Cell culture**

1. From a glycerol stock, streak a sample of YPH499 yeast cells on a YPD plate and grow at 30 °C for 2 days.
2. Collect the cells from the plate using a disposable loop, inoculate a 150 mL culture of YPD medium in a 500 mL flask with the cells, and then, grow overnight (approximately 16 h) at 30 °C with vigorous shaking at 170 rpm.
3. Inoculate six 900 mL culture of YPD medium in 2 L baffled flasks with 20 mL of the overnight culture per flask and grow at 30 °C with vigorous shaking at 170 rpm until the culture reaches an OD_600_ of 4.0. This should take approximately 9 h.
4. Harvest the cells by centrifugation at 9000× *g* for 10 min at 4 °C.
5. Resuspend with 1 L of 1% (wt/vol) KCl and harvest the cells by centrifugation at 9000× *g* for 10 min at 4 °C.
6. Resuspend with 1 L of lysis buffer and harvest the cells by centrifugation at 9000× *g* for 10 min at 4 °C. The pellet weight should be 4.0 g/L culture.
7. Resuspend with an equal amount of lysis buffer (1 mL buffer per 1 g cells) and dispense the cells, through micropipettes, into liquid nitrogen to make frozen yeast droplets.

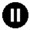
**PAUSE STEP** Frozen cells can be stored at −80 °C for approximately one month.

**Cell lysis**

1. Disrupt the cells by mechanical grinding (see Appendix A).
2. Thaw the frozen powder of ground cells at 4 °C and centrifuge at 7500× *g* for 10 min at 4 °C. Thereafter, transfer the supernatant to a clean tube (Sup 1, approximately 20 mL).
3. Resuspend the resulting pellets with 30 mL of lysis buffer and centrifuge at 7500× *g* for 10 min at 4 °C. Thereafter, transfer the supernatant to a clean tube (Sup 2, approximately 30 mL).
4. Combine Sup 1 and Sup 2 and transfer to a clean ultracentrifuge tube. Centrifuge at 150,000× *g* (450,000 rpm, Type 70Ti, BECKMAN COULTER) for 3 h at 4 °C. Thereafter, transfer the supernatants to a clean tube (S100, approximately 40 mL).

**NOTE:** Take care to minimize contamination from either the lipid fraction at the very top or the cell debris at the bottom of the tube.

**Batch purification with Q sepharose**

1. Incubate S100 and 15 mL of Q sepharose Fast Flow resin (Cytiva) pre-equilibrated with lysis buffer on a rotator at 4 °C for approximately 30 min.
2. Sediment the resin by centrifugation at 500× *g* for 10 min, then transfer the supernatant to a clean tube (flow-through fraction, approximately 40 mL).
3. Add 1 column volume of lysis buffer to the resin and gently mix the slurry. Sediment the resin by centrifugation at 500× *g* for 10 min, and then, transfer the supernatant to a clean tube (wash fraction, approximately 15 mL).
4. Combine the flow-through and wash fractions (approximately 55 mL).

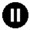
**PAUSE STEP** Freeze the sample with liquid nitrogen; then, it can be stored at −80 °C for approximately 1 week.

**Purification by HiTrap SP column chromatography**

1. Load the sample (55 mL) at a flow rate of 1.0 mL/min onto a HiTrap SP HP column (10 mL, Cytiva) that has been equilibrated with 5% (50 mM KCl) SP B buffer.
2. After washing with 5 column volumes of 10% (100 mM KCl) SP B buffer, elute with a linear gradient from 10 to 22.2% (100 to 220 mM KCl) SP B buffer in 5 column volumes at a flow rate of 1.0 mL/min, collecting 2 mL fractions.
3. Elute with another 5 column volumes of 22.2% (220 mM KCl) SP B buffer.
4. Analyze the fractions by 10% SDS-PAGE and pool fractions containing eEF1A. Typically, eEF1A is eluted at around 22% SP B buffer, the volume of the pool is approximately 15 mL.

**Purification by HiTrap Butyl column chromatography**

1. Add solid (NH_4_)_2_SO_4_ slowly to the eEF1A pool, to a final concentration of 1.4 M (0.185 g per 1 mL of eEF1A pool), mixing gently with a magnetic stirrer at 4 °C.
2. Centrifuge the sample at 10,000× *g* for 10 min at 4 °C. Thereafter, transfer the supernatant to a clean tube.
3. Load the sample at a flow rate of 1.0 mL/min onto a HiTrap Butyl FF column (10 mL, Cytiva) which has been equilibrated with 0% (1.4 M (NH_4_)_2_SO_4_) Butyl B buffer.
4. After washing with 5 column volumes of 40% (840 mM (NH_4_)_2_SO_4_) Butyl B buffer, elute with a linear gradient from 40 to 100% (840 to 0 mM (NH_4_)_2_SO_4_) Butyl B buffer in 15 column volumes at a flow rate of 1.0 mL/min, collecting 2 mL fractions.
5. Analyze the fractions by 10% SDS-PAGE and pool fractions that contain eEF1A. Typically, eEF1A is eluted at about 65% Butyl B buffer as a major peak, and the volume of the pool is approximately 30 mL. Be careful not to collect the base of the peak, which contain impurities.
6. Dialyze the sample against 3 L of eEF1A stock buffer at 4 °C overnight with two changes of buffer; dialyze 2 h, 16 h, and another 2 h.

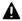
** CRITICAL STEP** Residual ammonium sulfate in the purified eEF1A inhibits the translation.

7. Concentrate the purified protein to approximately 5 mg/mL using Amicon Ultra Centrifugal Filters (10 kDa cut-off) (MERCK).
8. Determine the concentration of the protein by the Bradford Assay or by using the adsorption coefficient of eEF1A (Figure S1).
9. The purified factors are aliquoted, fast-frozen in liquid nitrogen, and stored at −80 °C. The yield is about 1 mg/L culture (Table S2).

**Notes**

1. To check the activity of the purified eEF1A, it is recommended to perform the translation reaction both in the presence and absence of eEF3, in order to analyze the eEF3 contamination levels. The purified eEF1A is contaminated with a small amount of eEF3 (<1%), as estimated by western blotting analysis. Note here that a high concentration of eEF1A (10–20 times the ribosomal concentration) is utilized in a standard translation reaction condition. Therefore, only when extremely pure eEF1A is obtained, the omission of eEF3 reduces the translation efficiency by about half. Otherwise, there is little effect of eEF3 on the translation efficiency. In our hands, further purification by the gel filtration do not eliminate the contaminating eEF3 from the purified eEF1A.

#### 3.1.2. eEF2

**Cell culture**

1. From a glycerol stock, streak a sample of TKY675/p416GPD-eEF2 yeast cells on a YPD plate and grow at 30 °C for 2 days.
2. Collect the cells from the plate using a disposable loop, inoculate a 300 mL culture of YPD medium in a 500 mL flask with the cells, and then grow overnight (approximately 16 h) at 30 °C with vigorous shaking at 170 rpm.
3. Inoculate twelve 900 mL culture of YPD medium in 2 L baffled flasks with 20 mL of the overnight culture per flask and grow at 30 °C with vigorous shaking at 170 rpm, until the culture reaches an OD_600_ of 4.0. This should take approximately 24 h.
4. Harvest the cells by centrifugation at 9000× *g* for 10 min at 4 °C.
5. Resuspend with 1 L of 1% (wt/vol) KCl and harvest the cells by centrifugation at 9000× *g* for 10 min at 4 °C.
6. Resuspend with 1 L of 1% (wt/vol) KCl and harvest the cells by centrifugation at 9000× *g* for 10 min at 4 °C. The pellet weight should be 4.8 g/L culture.

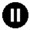
** PAUSE STEP** Frozen cells can be stored at −80 °C for approximately one month.

**Cell lysis**

1. Add 2.5 mL of lysis buffer (Y-PER containing 10 μM GDP, 0.2 mM PMSF, and 5 mM DTT) per 1 g of cells.
2. Stir until the mixture is homogenous, then stir for 20 min at room temperature.
3. Centrifuge the samples at 7500× *g* for 10 min at 4 °C and keep the supernatants.

**Purification with Ni-NTA agarose**

1. Incubate the lysate and 2 mL of Ni-NTA agarose resin (QIAGEN) equilibrated with Ni-NTA wash Buffer I on a rotator at 4 °C for approximately 30 min.
2. Load the slurry into empty columns (1 mL resin/column, 2 columns). Collect the flow-through fractions in a clean tube.
3. Wash with 20 column volumes of Ni-NTA wash Buffer I (20 mL buffer/column). Collect the wash fractions into clean tubes.
4. Wash with 20 column volumes of Ni-NTA wash Buffer II (20 mL buffer/column). Collect the wash fractions into clean tubes.
5. Wash with 30 column volumes of Ni-NTA wash Buffer I (30 mL buffer/column). Collect the wash fractions into clean tubes.
6. Elute with 4 column volumes of Ni-NTA elution buffer (4 mL buffer/column). Collect the eluate fractions into clean tubes.
7. Analyze the fractions by 10% SDS-PAGE and pool fractions containing eEF2.
8. Dialyze the sample against 1 L of dialysis buffer at 4 °C overnight with a change of buffer after 1 h.
9. **OPTIONAL STEP:** Clarify the sample by centrifugation at 10,000× *g* for 15 min at 4 °C.

**Purification by HiTrap Q column chromatography**

1. Load the sample at a flow rate of 1.0 mL/min onto a Hitrap Q HP column (5 mL, Cytiva) which has been equilibrated with 10% (100 mM KCl) B buffer.
2. After washing with 5 column volumes of 10% (100 mM KCl) Q B buffer, elute with a linear gradient from 10 to 25% (100 to 250 mM KCl) Q B buffer in 15 column volumes at a flow rate of 2.0 mL/min, collecting 1.0 mL fractions.
3. Analyze the fractions by 10% SDS-PAGE and pool fractions that contain eEF2. Typically, eEF2 is eluted at about 23% B buffer as a major peak, and the volume of the pool is approximately 8 mL. Be careful not to collect the base of the peak, which contains impurities.
4. Dialyze the sample against 1 L of eEF2 stock buffer at 4 °C overnight, with a change of buffer after 1 h.
5. Concentrate the purified protein to approximately 5 mg/mL using Amicon Ultra Centrifugal Filters (10 kDa cut-off) (MERCK).
6. Determine the concentration of the protein by the Bradford Assay or by using the adsorption coefficient of eEF2 (Figure S1).
7. The purified factors are aliquoted, fast-frozen in liquid nitrogen, and stored at −80 °C. The yield is about 40 μg/L culture (Table S2).

#### 3.1.3. eEF3

**Cell culture**

1. Freshly transform the plasmid pET29b-eEF3 (Table S2) into competent JM109(DE3)pRARE *E. coli* cells and grow overnight (approximately 12–16 h) at 37 °C on LB plates supplemented with 25 μg/mL kanamycin, 25 μg/mL chloramphenicol, and 1% (wt/vol) glucose.
2. Pick a single colony and inoculate it into 50 mL of LB supplemented with 25 μg/mL kanamycin, 25 μg/mL chloramphenicol, and 1% (wt/vol) glucose, then grow overnight at 37 °C with vigorous shaking at 170 rpm.
3. Inoculate four 1 L cultures of 2 × YT supplemented with 25 μg/mL kanamycin, 25 μg/mL chloramphenicol, and 0.1% (wt/vol) glucose in 2 L baffled flasks with 10 mL of the overnight culture per flask and grow at 37 °C with vigorous shaking at 170 rpm, until the culture reaches an OD_600_ of 0.5. This should take approximately 2 h.
4. Induce protein expression by adding 1 mL of 100 mM IPTG (final 0.1 mM) per flask and grow at 37 °C for 2 h with vigorous shaking at 170 rpm.
5. Harvest the cells by centrifugation at 9000× *g* for 10 min at 4 °C.
6. Resuspend with 1 L of 1% (wt/vol) KCl and harvest the cells by centrifugation at 9000× *g* for 10 min at 4 °C.
7. Resuspend with 1 L of lysis buffer and harvest the cells by centrifugation at 9000× *g* for 10 min at 4 °C. The pellet weight should be 2.0 g/L culture.

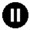
** PAUSE STEP** Frozen cells can be stored at −80 °C for approximately one month.

**Cell lysis**

1. Disrupt the cells by sonication (see Appendix A).
2. Centrifuge the disrupted cells at 10,000× *g* for 30 min at 4 °C. Thereafter, transfer the supernatants to a clean tube.
3. Centrifuge the sample again at 10,000× *g* for 30 min at 4 °C. Thereafter, transfer the supernatants to a clean tube.

**Purification with Ni-NTA agarose**

1. Incubate the lysate and 2 mL of Ni-NTA agarose resin (QIAGEN) equilibrated with lysis buffer on a rotator at 4 °C for approximately 30 min.
2. Load the slurry into empty columns (1 mL resin/column, 2 columns). Collect the flow-through fractions in clean tubes.
3. Wash with 100 column volumes of Ni-NTA wash buffer (100 mL buffer/column). Collect the wash fractions and transfer to clean tubes.
4. Elute with 5 column volumes of Ni-NTA elution buffer (5 mL buffer/column). Collect the eluate fractions and transfer to clean tubes.
5. Analyze the fractions by 10% SDS-PAGE and pool fractions containing eEF3.
6. Dialyze the sample against 1 L of dialysis buffer at 4 °C overnight with a change of buffer after 1 h.
7. **OPTIONAL STEP:** Clarify the sample by centrifugation at 10,000× *g* for 15 min at 4 °C.

**Purification by HiTrap Q column chromatography**

1. After diluting the sample 2-fold by adding an equal volume of 0% (0 mM KCl) Q B buffer, load the sample at a flow rate of 0.5 mL/min onto a HiTrap Q column (5 mL, Cytiva) which has been equilibrated with 5% (50 mM KCl) B buffer.
2. After washing with 5 column volumes of 5% (50 mM KCl) Q B buffer, elute with a linear gradient from 5 to 35% (50 to 350 mM KCl) Q B buffer in 15 column volumes at a flow rate of 0.5 mL/min, collecting 1 mL fractions.
3. Analyze the fractions by 10% SDS-PAGE and pool fractions that contain eEF3. Typically, eEF3 is eluted at about 17% Q B buffer as a major peak, and the volume of the pool is approximately 7 mL. Be careful not to collect the base of the peak, which contains impurities.
4. Dialyze the sample against 1 L of eEF3 stock buffer at 4 °C overnight, with a change of buffer after 1 h.
5. Concentrate the purified protein to approximately 5 mg/mL using Amicon Ultra Centrifugal Filters (10 kDa cut-off) (MERCK).
6. Determine the concentration of the protein by the Bradford Assay or by using the adsorption coefficient of eEF3 (Figure S1).
7. The purified factors are aliquoted, fast-frozen in liquid nitrogen, and stored at −80 °C. The yield is about 700 μg/L culture (Table S2).

#### 3.1.4. eRF1

**Cell culture**

1. Freshly transform the plasmid pET21a-eRF1 (Table S2) into competent Rosetta(DE3)pLysS *E. coli* cells and grow overnight (approximately 12–16 h) at 37 °C on LB plates supplemented with 100 μg/mL ampicillin, 25 μg/mL chloramphenicol, and 1% (wt/vol) glucose.
2. Pick a single colony and inoculate it into 30 mL of LB supplemented with 100 μg/mL ampicillin, 25 μg/mL chloramphenicol, and 1% (wt/vol) glucose, and then, grow overnight at 37 °C with vigorous shaking at 170 rpm.
3. Inoculate two 1 L cultures of 2 × YT supplemented with 100 μg/mL ampicillin and 25 μg/mL chloramphenicol with 10 mL of the overnight culture per flask, and then, grow at 37 °C with vigorous shaking at 170 rpm until the culture reaches an OD_600_ of 0.5. This should take approximately 2 h.
4. Induce protein expression by adding 1 mL of 100 mM IPTG (final 0.1 mM) per flask and grow overnight (approximately 12–16 h) at 18 °C with vigorous shaking at 170 rpm.
5. Harvest the cells by centrifugation at 9000× *g* for 10 min at 4 °C.
6. Resuspend with 1 L of 1% (wt/vol) KCl and harvest the cells by centrifugation at 9000× *g* for 10 min at 4 °C.
7. Resuspend with 1 L of lysis buffer and harvest the cells by centrifugation at 9000× *g* for 10 min at 4 °C. The pellet weight should be 8.0 g/L culture.

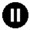
** PAUSE STEP** Frozen cells can be stored at −80 °C for approximately one month.

**Cell lysis**

1. Disrupt the cells by sonication (see Appendix A).
2. Centrifuge the disrupted cells at 10,000× *g* for 30 min at 4 °C. Thereafter, transfer the supernatants to a clean tube.
3. Centrifuge the sample again at 10,000× *g* for 30 min at 4 °C. Thereafter, transfer the supernatants to a clean tube.

**Purification with Ni-NTA agarose**

1. Incubate the lysate and 2 mL of Ni-NTA agarose resin (QIAGEN) equilibrated with lysis buffer on a rotator at 4 °C for approximately 30 min.
2. Load the slurry into empty columns (1 mL resin/column, 2 columns). Collect the flow-through fractions in clean tubes.
3. Wash with 100 column volumes of Ni-NTA wash buffer (100 mL buffer/column). Collect the wash fractions in clean tubes.
4. Elute with 5 column volumes of Ni-NTA elution buffer (5 mL buffer/column). Collect the eluate fractions in clean tubes.
5. Analyze the fractions by 10% SDS-PAGE and pool fractions containing eRF1.
6. Dialyze the sample against 1 L of stock buffer at 4 °C overnight, with a change of buffer after 1 h.
7. **OPTIONAL STEP:** Clarify the sample by centrifugation at 10,000× *g* for 15 min at 4 °C.

**Purification by HiTrap Q column chromatography**

1. Load the sample at a flow rate of 1.0 mL/min onto a HiTrap Q HP column (20 mL, Cytiva) that has been equilibrated with 10% (100 mM KCl) B buffer.
2. Wash with 5 column volumes of 20% (200 mM KCl) Q B buffer, then with another 5 column volumes of 30% (300 mM KCl) Q B buffer, at a flow rate of 2.0 mL/min.
3. Elute with a linear gradient from 30 to 60% (300 to 600 mM KCl) B buffer in 15 column volumes at a flow rate of 2.0 mL/min, collecting 4 mL fractions.
4. Analyze the fractions by 10% SDS-PAGE and pool fractions that contain eRF1. Typically, eRF1 is eluted at about 36% B buffer as a major peak, and the volume of the pool is approximately 20 mL. Be careful not to collect the base of the peak, which contains impurities.
5. Dialyze the sample against 1 L of eRF1 stock buffer at 4 °C overnight, with a change of buffer after 1 h.
6. Concentrate the purified protein to approximately 5 mg/mL using Amicon Ultra Centrifugal Filters (10 kDa cut-off) (MERCK).
7. Determine the concentration of the protein by the Bradford Assay or by using the adsorption coefficient of eRF1 (Figure S1).
8. The purified factors are aliquoted, fast-frozen in liquid nitrogen, and stored at −80 °C. The yield is about 8.5 mg/L culture (Table S2).

#### 3.1.5. eRF3∆165

**Cell culture**

1. Freshly transform the plasmid pET21a-eRF3∆165 (Table S2) into competent BL21(DE3) *E. coli* cells and grow overnight (approximately 12–16 h) at 37 °C on LB plates supplemented with 100 μg/mL ampicillin and 1% (wt/vol) glucose.
2. Pick a single colony and inoculate it into 30 mL of LB supplemented with 100 μg/mL ampicillin, then grow overnight at 37 °C with vigorous shaking at 170 rpm.
3. Inoculate two 1 L cultures of 2 x YT supplemented with 100 μg/mL ampicillin with 10 mL of the overnight culture per flask, then grow at 37 °C with vigorous shaking at 170 rpm, until the culture reaches an OD_600_ of 0.5. This should take approximately 2 h.
4. Induce protein expression by adding 1 mL of 100 mM IPTG (final 0.1 mM) per flask and grow overnight (approximately 12–16 h) at 18 °C with vigorous shaking at 170 rpm.
5. Harvest the cells by centrifugation at 9000× *g* for 10 min at 4 °C.
6. Resuspend with 1 L of 1% (wt/vol) KCl and harvest the cells by centrifugation at 9000× *g* for 10 min at 4 °C.
7. Resuspend with 1 L of lysis buffer and harvest the cells by centrifugation at 9000× *g* for 10 min at 4 °C. The pellet weight should be 7.8 g/L culture.

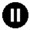
** PAUSE STEP** Frozen cells can be stored at −80 °C for approximately one month.

**Cell lysis**

1. Disrupt the cells by sonication (see Appendix A).
2. Centrifuge the disrupted cells at 10,000× *g* for 30 min at 4 °C. Thereafter, transfer the supernatants to a clean tube.
3. Centrifuge the sample again at 10,000× *g* for 30 min at 4 °C. Thereafter, transfer the supernatants to a clean tube.

**Purification with Ni-NTA agarose**

1. Incubate the lysate and 4 mL of Ni-NTA agarose resin (QIAGEN) equilibrated with lysis buffer on a rotator at 4 °C for approximately 30 min.
2. Load the slurry into empty columns (1 mL resin/column, 4 columns). Collect the flow-through fractions in clean tubes.
3. Wash with 100 column volumes of Ni-NTA wash buffer (100 mL buffer/column). Collect the wash fractions in clean tubes.
4. Elute with 5 column volumes of Ni-NTA elution buffer (5 mL buffer/column). Collect the eluate fractions in clean tubes.
5. Analyze the fractions by 10% SDS-PAGE and pool fractions containing eRF3∆165.
6. Dialyze the sample against 1 L of stock buffer at 4 °C overnight, with a change of buffer after 1 h.
7. **OPTIONAL STEP:** Clarify the sample by centrifugation at 10,000× *g* for 15 min at 4 °C.

**Purification by HiTrap Q column chromatography**

1. Load the sample at a flow rate of 1.0 mL/min onto a HiTrap Q HP column (20 mL, Cytiva) which has been equilibrated with 10% (100 mM KCl) Q B buffer.
2. After washing with 5 column volumes of 10% (100 mM KCl) Q B buffer, elute with a linear gradient from 10 to 30% (100 to 300 mM KCl) Q B buffer in 15 column volumes at a flow rate of 2.0 mL/min, collecting 4 mL fractions.
3. Analyze the fractions by 10% SDS-PAGE and pool fractions that contain eRF3∆165. Typically, eRF3∆165 is eluted at about 17% B buffer as a major peak, and the volume of the pool is approximately 35 mL. Be careful not to collect the base of the peak, which contains impurities.
4. Dialyze the sample against 2 L of eRF3∆165 stock buffer at 4 °C overnight, with a change of buffer after 1 h.
5. Concentrate the purified protein to approximately 5 mg/mL using Amicon Ultra Centrifugal Filters (10 kDa cut-off) (MERCK).
6. Determine the concentration of the protein by the Bradford Assay or by using the adsorption coefficient of eRF3 (Figure S1).
7. The purified factors are aliquoted, fast-frozen in liquid nitrogen, and stored at −80 °C. The yield is about 24 mg/L culture (Table S2).

**Notes**

1. eRF3∆165 is the more stable version of the full-length eRF3 with the deletion of the N-terminal amino acids not involved in function.

#### 3.1.6. Dom34

**Cell culture**

1. Freshly transform the plasmid pET29b-Dom34 (Table S2) into competent Rosetta(DE3)pLysS *E. coli* cells and grow overnight (approximately 12–16 h) at 37 °C on LB plates supplemented with 25 μg/mL kanamycin, 25 μg/mL chloramphenicol, and 1% (wt/vol) glucose.
2. Pick a single colony and inoculate it into 30 mL of LB supplemented with 25 μg/mL kanamycin, 25 μg/mL chloramphenicol, and 1% (wt/vol) glucose, then grow overnight at 37 °C with vigorous shaking at 170 rpm.
3. Inoculate two 1 L cultures of 2 × YT supplemented with 25 μg/mL kanamycin and 25 μg/mL chloramphenicol with 10 mL of the overnight culture per flask, and then, grow at 37 °C with vigorous shaking at 170 rpm, until the culture reaches an OD_600_ of 0.5. This should take approximately 2 h.
4. Induce protein expression by adding 1 mL of 100 mM IPTG (final 0.1 mM) per flask and grow overnight (approximately 12–16 h) at 18 °C with vigorous shaking at 170 rpm.
5. Harvest the cells by centrifugation at 9000× *g* for 10 min at 4 °C.
6. Resuspend with 1 L of 1% (wt/vol) KCl and harvest the cells by centrifugation at 9000× *g* for 10 min at 4 °C.
7. Resuspend with 1 L of lysis buffer and harvest the cells by centrifugation at 9000× *g* for 10 min at 4 °C. The pellet weight should be 9.6 g/L culture.

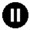
** PAUSE STEP** Frozen cells can be stored at −80 °C for approximately one month.

**Cell lysis**

1. Disrupt the cells by sonication (see Appendix A).
2. Centrifuge the disrupted cells at 10,000× *g* for 30 min at 4 °C. Thereafter, transfer the supernatants to a clean tube.
3. Centrifuge the sample again at 10,000× *g* for 30 min at 4 °C. Thereafter, transfer the supernatants to a clean tube.

**Purification with Ni-NTA agarose**

1. Incubate the lysate and 4 mL of Ni-NTA agarose resin (QIAGEN) equilibrated with lysis buffer on a rotator at 4 °C for approximately 30 min.
2. Load the slurry into empty columns (1 mL resin/column, 4 columns). Collect the flow-through fractions in clean tubes.
3. Wash with 100 column volumes of Ni-NTA wash buffer (100 mL buffer/column). Collect the wash fractions in clean tubes.
4. Elute with 5 column volumes of Ni-NTA elution buffer (5 mL buffer/column). Collect the eluate fractions in clean tubes.
5. Analyze the fractions by 10% SDS-PAGE and pool fractions containing Dom34.
6. Dialyze the sample against 1 L of stock buffer at 4 °C overnight, with a change of buffer after 1 h.
7. **OPTIONAL STEP:** Clarify the sample by centrifugation at 10,000× *g* for 15 min at 4 °C.

**Purification by HiPrep Q column chromatography**

1. After diluting the sample 2-fold by adding an equal volume of 0% (0 mM KCl) Q B buffer, load the sample at a flow rate of 2.0 mL/min onto a HiPrep Q HP column (20 mL, Cytiva) which has been equilibrated with 10% (100 mM KCl) Q B buffer.
2. After washing with 5 column volumes of 10% (100 mM KCl) Q B buffer, elute with a linear gradient from 10 to 50% (100 to 500 mM KCl) Q B buffer in 15 column volumes at a flow rate of 2.0 mL/min, collecting 4 mL fractions.
3. Analyze the fractions by 10% SDS-PAGE and pool fractions that contain Dom34. Typically, Dom34 is eluted as a major peak at about 24% Q B buffer, with the former half of the peak containing impurities of about 38 kDa and the latter half of the peak containing impurities of about 40 kDa. Pool the latter half of the peak, which is approximately 50 mL.
4. Dialyze the sample against 2 L of Dom34 stock buffer at 4 °C overnight with a change of buffer after 1 h.

**Purification by HiLoad 16/600 Superdex 200 pg column chromatography**

1. Concentrate the sample volume to ≤5 mL using Amicon Ultra Centrifugal Filters (10 kDa cut-off) (MERCK).
2. Load the sample at a flow rate of 0.5 mL/min onto a HiLoad 16/600 Superdex 200 pg column (Cytiva) which has been equilibrated with Dom34 stock buffer, collecting 1.2 mL fractions.
3. Analyze the fractions by 10% SDS-PAGE and pool fractions that contain Dom34. Typically, Dom34 is eluted at around 70 mL as a major peak, and the volume of the pool is approximately 7 mL. Be careful not to collect the base of the peak, which contains impurities.
4. Concentrate the purified protein to approximately 5 mg/mL using Amicon Ultra Centrifugal Filters (10 kDa cut-off) (MERCK).
5. Determine the concentration of the protein by the Bradford Assay or by using the adsorption coefficient of Dom34 (Figure S1).
6. The purified factors are aliquoted, fast-frozen in liquid nitrogen, and stored at −80 °C. The yield is about 2.2 mg/L culture (Table S2).

#### 3.1.7. Hbs1

**Cell culture**

1. Freshly transform the plasmid pETDuet-1-Hbs1 (Table S2) into competent BL21(DE3) *E. coli* cells, and then, grow overnight (approximately 12–16 h) at 37 °C on LB plates supplemented with 100 μg/mL ampicillin and 1% (wt/vol) glucose.
2. Pick a single colony and inoculate it into 30 mL of LB supplemented with 100 μg/mL ampicillin, and then, grow overnight at 37 °C with vigorous shaking at 170 rpm.
3. Inoculate two 1 L cultures of 2 × YT supplemented with 100 μg/mL ampicillin with 10 mL of the overnight culture per flask, and then grow at 37 °C with vigorous shaking at 170 rpm, until the culture reaches an OD_600_ of 0.5. This should take approximately 2 h.
4. Induce protein expression by adding 1 mL of 100 mM IPTG (final 0.1 mM) per flask, and then, grow overnight (approximately 12–16 h) at 18 °C with vigorous shaking at 170 rpm.
5. Harvest the cells by centrifugation at 9000× *g* for 10 min at 4 °C.
6. Resuspend with 1 L of 1% (wt/vol) KCl and harvest the cells by centrifugation at 9000× *g* for 10 min at 4 °C.
7. Resuspend with 1 L of lysis buffer and harvest the cells by centrifugation at 9000× *g* for 10 min at 4 °C. The pellet weight should be 7.8 g/L culture.

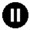
** PAUSE STEP** Frozen cells can be stored at −80 °C for approximately one month.

**Cell lysis**

1. Disrupt the cells by sonication (see Appendix A).
2. Centrifuge the disrupted cells at 10,000× *g* for 30 min at 4 °C. Thereafter, transfer the supernatants to a clean tube.
3. Centrifuge the sample again at 10,000× *g* for 30 min at 4 °C. Thereafter, transfer the supernatants to a clean tube.

**Purification with Ni-NTA agarose**

1. Incubate the lysate and 2 mL of Ni-NTA agarose resin (QIAGEN) equilibrated with lysis buffer on a rotator at 4 °C for approximately 30 min.
2. Load the slurry into empty columns (1 mL resin/column, 2 columns). Collect the flow-through fractions in clean tubes.
3. Wash with 100 column volumes of Ni-NTA wash buffer (100 mL buffer/column). Collect the wash fractions in clean tubes.
4. Elute with 5 column volumes of Ni-NTA elution buffer (5 mL buffer/column). Collect the eluate fractions in clean tubes.
5. Analyze the fractions by 10% SDS-PAGE and pool fractions containing Hbs1.
6. Dialyze the sample against 1 L of stock buffer at 4 °C overnight, with a change of buffer after 1 h.
7. **OPTIONAL STEP:** Clarify the sample by centrifugation at 10,000× *g* for 15 min at 4 °C.

**Purification by HiTrap Q column chromatography**

1. Load the sample at a flow rate of 1 mL/min onto a HiTrap Q column (10 mL, Cytiva) which has been equilibrated with 10% (100 mM KCl) Q B buffer.
2. After washing with 3 column volumes of 10% (100 mM KCl) Q B buffer and then with another 3 column volumes of 25% (250 mM KCl) Q B buffer, elute with a linear gradient from 25 to 60% (250 to 600 mM KCl) Q B buffer in 15 column volumes at a flow rate of 2 mL/min, collecting 2 mL fractions.
3. Analyze the fractions by 10% SDS-PAGE and pool fractions that contain Hbs1. Typically, Hbs1 is eluted at about 37% Q B buffer as a major peak, and the volume of the pool is approximately 10 mL. Be careful not to collect the base of the peak, which contains impurities.
4. Dialyze the sample against 1 L of Hbs1 stock buffer at 4 °C overnight, with a change of buffer after 1 h.
5. Concentrate the purified protein to approximately 5 mg/mL using Amicon Ultra Centrifugal Filters (10 kDa cut-off) (MERCK).
6. Determine the concentration of the protein by the Bradford Assay or by using the adsorption coefficient of Hbs1 (Figure S1).
7. The purified factors are aliquoted, fast-frozen in liquid nitrogen, and stored at −80 °C. The yield is about 1.6 mg/L culture (Table S2).

#### 3.1.8. Rli1

**Cell culture**

1. From a glycerol stock, streak a sample of INVSc1/pYES2-Rli1 yeast cells on a SC-Ura plate containing 1% (wt/vol) raffinose, and then, grow at 30 °C for 2 days.
2. Collect the cells from the plate using a disposable loop, inoculate two 400 mL cultures of SC-Ura medium containing 1% (wt/vol) raffinose in 500 mL flasks with the cells, and then grow at 30 °C for 2 days with vigorous shaking at 170 rpm.
3. Inoculate twelve 720 mL cultures of SC-Ura medium containing 1% (wt/vol) raffinose in 2 L baffled flasks with 60 mL of the overnight culture per flask, and then, grow at 30 °C for 2 h with vigorous shaking at 170 rpm.
4. Induce protein expression by adding 80 mL of 20% (wt/vol) galactose (final concentration 2% (wt/vol)) per flask, and then, grow at 30 °C with vigorous shaking at 170 rpm, until the culture reaches an OD_600_ of 7.0. This should take approximately 20 h.
5. Harvest the cells by centrifugation at 9000× *g* for 10 min at 4 °C.
6. Resuspend with 1 L of 1% (wt/vol) KCl and harvest the cells by centrifugation at 9000× *g* for 10 min at 4 °C.
7. Resuspend with 1 L of lysis buffer and harvest the cells by centrifugation at 9000× *g* for 10 min at 4 °C. The pellet weight should be 4.0 g/L culture.
8. Resuspend with an equal amount of lysis buffer (1 mL buffer per 1 g cells) and dispense the cells through micropipettes into liquid nitrogen, to form frozen yeast droplets.

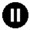
** PAUSE STEP** Frozen cells can be stored at −80 °C for approximately one month.

**Cell lysis**

1. Disrupt the cells by mechanical grinding (see Appendix A).
2. Add 25 mL of lysis buffer to the frozen powder of ground cells and thaw the sample at 4 °C. Thereafter, centrifuge at 7500× *g* for 10 min at 4 °C, and then, transfer the supernatants to a clean tube (Sup 1, approximately 80 mL).
3. Resuspend the resulting pellets with 75 mL of lysis buffer.
4. Disrupt the suspension of cell debris again using a sonicator (Branson) (pulse on, 2 s; pulse off, 8 s; power, 80 watts; time, 5 min).
5. Centrifuge the sample at 7500× *g* for 10 min at 4 °C. Thereafter, transfer the supernatants to a clean tube (Sup 2, approximately 50 mL).
6. Combine Sup 1 and Sup 2 and transfer to a clean ultracentrifuge tube. Centrifuge at 17,000× *g* (15,000 rpm, Type 45Ti, BECKMAN COULTER) for 30 min at 4 °C. Thereafter, transfer the supernatants to a clean tube (approximately 110–120 mL).

**NOTE:** Take care to minimize contamination from either the lipid fraction at the very top or the cell debris at the bottom of the tube.

**Purification with Ni-NTA agarose**

1. Incubate the lysate and 2 mL of Ni-NTA agarose resin (QIAGEN) equilibrated with lysis buffer on a rotator at 4 °C for approximately 30 min.
2. Load the slurry into empty columns (1 mL resin/column, 2 columns). Collect the flow-through fractions in clean tubes.
3. Wash with 100 column volumes of Ni-NTA wash buffer (100 mL buffer/column). Collect the wash fractions in clean tubes.
4. Wash with 50 column volumes of Ni-NTA wash buffer containing 1% Tween 20 (50 mL buffer/column). Collect the wash fractions in clean tubes.
5. Elute with 5 column volumes of Ni-NTA elution buffer (5 mL buffer/column). Collect the eluate fractions in clean tubes.
6. Analyze the fractions by 10% SDS-PAGE and pool fractions containing Rli1.
7. Dialyze the sample against 1 L of HiTrap SP A buffer containing 0.1 mM PMSF at 4 °C overnight, with a change of buffer after 1 h.
8. **OPTIONAL STEP:** Clarify the sample by centrifugation at 10,000× *g* for 15 min at 4 °C.

**Purification by Q sepharose column chromatography**

1. Incubate the sample and 3 mL of Q sepharose Fast Flow resin (Cytiva) equilibrated with HiTrap SP A buffer on a rotator at 4 °C for approximately 30 min.
2. Sediment the resin by centrifugation at 500× *g* for 10 min, and then, transfer the supernatant to a clean tube (flow-through Fraction 1, approximately 45 mL).
3. Add 1 column volume of HiTrap SP A buffer to the resin and gently mix the slurry. Thereafter, sediment the resin by centrifugation at 500× *g* for 10 min, and then, transfer the supernatant to a clean tube (flow-through Fraction 2, approximately 3 mL).
4. Combine the flow-through Fractions 1 and 2 in a clean tube (approximately 50 mL).

**Purification by HiTrap SP column chromatography**

1. Load the sample at a flow rate of 1.0 mL/min onto a HiTrap SP HP column (5 mL, Cytiva) which has been equilibrated with 0% (100 mM KCl) SP B buffer.
2. After washing with 5 column volumes of 0% (100 mM KCl) SP B buffer, elute with a linear gradient from 0 to 40% (100 to 460 mM KCl) SP B buffer in 10 column volumes at a flow rate of 2.0 mL/min, collecting 1 mL fractions.
3. Analyze the fractions by 10% SDS-PAGE and pool fractions that contain Rli1. Typically, Rli1 is eluted at about 22% SP B buffer as a major peak, and the volume of the pool is approximately 8 mL. Be careful not to collect the base of the peak, which contains impurities.
4. Dialyze the sample against 1 L of Rli1 stock buffer at 4 °C overnight, with a change of buffer after 1 h.
5. Concentrate the purified protein to approximately 5 mg/mL using Amicon Ultra Centrifugal Filters (10 kDa cut-off) (MERCK).
6. Determine the concentration of the protein by the Bradford Assay or by using the adsorption coefficient of Rli1 (Figure S1).
7. The purified factors are aliquoted, fast-frozen in liquid nitrogen, and stored at −80 °C. The yield is about 35 μg/L culture (Table S2).

#### 3.1.9. Ribosomes

**Cell culture**

1. From a glycerol stock, streak a sample of W303 yeast cells on a YPD plate and grow at 30 °C for 1 day.
2. Collect the cells from the plate using a disposable loop, inoculate a 300 mL culture of YPD medium in a 500 mL flask with the cells, and then, grow overnight (approximately 16 h) at 30 °C with vigorous shaking at 170 rpm.
3. Inoculate twelve 900 mL cultures of YPD medium in 2 L baffled flasks with 20 mL of the overnight culture per flask, and then, grow at 30 °C with vigorous shaking at 170 rpm, until the culture reaches an OD_600_ of 1.0 ± 0.1. This should take approximately 4–6 h.
4. Harvest the cells by centrifugation at 9000× *g* for 10 min at 4 °C.
5. Resuspend with 1 L of 1% (wt/vol) KCl and harvest the cells by centrifugation at 9000× *g* for 10 min at 4 °C.
6. Resuspend with 1 L of lysis buffer and harvest the cells by centrifugation at 9000× *g* for 10 min at 4 °C. The pellet weight should be 1.0 g/L culture.
7. Resuspend with an equal amount of lysis buffer (1 mL buffer per 1 g cells) and dispense the cells through micropipettes into liquid nitrogen to form frozen yeast droplets.

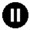
** PAUSE STEP** Frozen cells can be stored at −80 °C for approximately one month.

**Cell lysis**

1. Disrupt the cells by mechanical grinding (see Appendix A).
2. Add 15 mL of lysis buffer to the frozen powder of ground cells and thaw the sample at 4 °C. Thereafter, centrifuge at 1000× *g* for 10 min at 4 °C, and then, transfer the supernatants to a clean tube (Sup 1).
3. Resuspend the resulting pellets with 22.5 mL of lysis buffer and centrifuge at 1000× *g* for 10 min at 4 °C. Thereafter, transfer the supernatants to a clean tube (Sup 2).
4. Combine Sup 1 and Sup 2 (approximately 50 mL), add 1.25 μL of TURBO DNase (Thermo Fisher Scientific) per 10 mL of lysate, and then, divide into two clean ultracentrifuge tubes.
5. Centrifuge at 30,000× *g* (21,000 rpm, Type 70Ti, BECKMAN COULTER) for 30 min at 4 °C. Thereafter, transfer the supernatants to a clean tube (S30, approximately 40 mL).

**NOTE:** Take care to minimize contamination from either the lipid fraction at the very top or the cell debris at the bottom of the tube.

**Purification of crude ribosomes**

1. Dilute approximately 40 mL of S30 with lysis buffer up to 90 mL and divide into four 70Ti polycarbonate ultracentrifuge tubes (BECKMAN COULTER).
2. Place 2.5 mL of sucrose cushion Buffer I at the very bottom of the 70Ti tubes using a syringe.
3. Centrifuge at 100,000× *g* (60,000 rpm, Type 70Ti, BECKMAN COULTER) for 2 h at 4 °C. Then, aspirate off the supernatants.
4. Add 500 μL of puromycin treatment buffer per ultracentrifuge tube, dissolve the pellet with gentle shaking for 20 min at 4 °C, and then transfer to a clean tube.
5. Repeat Step 4 three times (1 h in total).
6. Centrifuge at 13,000× *g* for 5 min at 4 °C. Thereafter, transfer the supernatant to a clean tube (crude ribosomes, approximately 6 mL).
7. Quantify the crude ribosomes using a spectrometer. The yield is approximately 1500 A_260_ units.

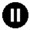
**PAUSE STEP** Freeze the sample with liquid nitrogen, they can be stored at −80 °C for approximately 1 day.

**Purification of 80S ribosomes by HiTrap Butyl column chromatography**

1. Add puromycin treatment buffer, such that the sample volume becomes 6 mL. Thereafter, add 300 μL of 10 mM puromycin (final 0.5 mM) and 60 μL of 100 mM GTP (final 1 mM) to the 6 mL of crude ribosomes; then, incubate the sample for 15 min at 27 °C.
2. Centrifuge at 13,000× *g* for 5 min at 4 °C. Thereafter, transfer the supernatant to a clean tube (puromycin-treated crude ribosomes, approximately 6 mL).
3. Add approximately 4 mL of Butyl A buffer to the puromycin-treated crude ribosomes, such that the sample volume becomes 10 mL. Then, slowly add 1.07 g of solid (NH_4_)_2_SO_4_ (final concentration 1.4 M), mixing gently with a magnetic stirrer at 4 °C.
4. Centrifuge the sample at 10,000× *g* for 10 min at 4 °C. Then, transfer the supernatant to a clean tube.
5. Load the sample at a flow rate of 1.0 mL/min onto a HiTrap Butyl FF column (20 mL, Cytiva) which has been equilibrated with 0% (1.4 M (NH_4_)_2_SO_4_) Butyl B buffer.
6. Wash the column, at a flow rate of 2.5 mL/min, through the four successive steps: (i) wash with 10 column volumes of 0% (1.4 M (NH_4_)_2_SO_4_) Butyl B buffer; (ii) wash with 5 column volumes of 30% (980 mM (NH_4_)_2_SO_4_) Butyl B buffer; (iii) wash with a linear gradient from 30 to 37% (980 to 882 mM (NH_4_)_2_SO_4_) Butyl B buffer in 5 column volumes; and (iv) wash with 37% B buffer with at least 10 column volumes, until the absorbance at 260 nm is near baseline.
7. Elute the ribosomes with a linear gradient from 37 to 50% (882 to 700 mM (NH_4_)_2_SO_4_) Butyl B buffer in 10 column volumes, and then with another 6.8 column volumes of 50% B buffer, at a flow rate of 2.5 mL/min, collecting 4 mL fractions.
8. Pool the fractions with A_260_ ≥ 2.

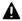
** CRITICAL STEP:** be careful not to pool the fractions of 60S subunits, which appear as the shoulder at the beginning of the major peak and whose A_260_ are usually < 2.

9. Transfer the 80S pool into a 45Ti polycarbonate ultracentrifuge tube (BECKMAN COULTER). Add 50% B buffer, such that the sample volume becomes 50 mL. If the volume of the 80S pool is greater than 50 mL, divide the sample into two 45Ti polycarbonate ultracentrifuge tubes and add 50% B buffer, such that the sample volume of each tube is 50 mL.
10. Place 20 mL of sucrose cushion Buffer II at the very bottom of the 45Ti tube(s) using a syringe.
11. Centrifuge at 100,000× *g* (36,000 rpm, Type 45Ti, BECKMAN COULTER) for 16 h at 4 °C. Thereafter, aspirate off the supernatants.
12. Add approximately 50 μL of 5/100 buffer per tube and dissolve the pellets by gently shaking for approximately 1 h at 4 °C, and then, transfer to clean tubes. Rinse the tube with a small amount of 5/100 buffer (approximately 10 μL) and combine it with the recovered ribosomes.
13. Measure the A_260_ of the sample using a spectrometer and determine the concentration of 80S ribosomes, estimating that the 80S ribosomes at 1.0 A_260_ correspond to 20 pmol.
14. The purified 80S ribosomes are aliquoted, fast-frozen in liquid nitrogen, and stored at −80 °C. The yield is about 110 pmol/L culture (Table S2).

**Analysis of ribosomal proteins**

1. Add 2.5 μL of 5× LiDS dye to 10 pmol of the purified 80S ribosomes in 10 μL of 5/100 buffer and mix well.
2. Apply the sample to a 15% SDS-PAGE gel, and then, visualize the protein bands by CBB R-250 staining.

**Analysis of ribosomal RNAs**

1. Mix 10 pmol of the purified 80S ribosomes, 250 μL of RNase-free water, 25 μL of 10% (wt/vol) SDS (final 1%), and 2.5 μL of 1 mg/mL of Proteinase K solution (final 10 μg/mL).
2. Incubate for 15 min at 37 °C.
3. Add 750 μL of ISOGEN-LS (NIPPON GENE) and vortex well.
4. After the incubation at room temperature for 5 min, add 200 μL of chloroform to the sample, and then, vortex well.
5. After the incubation at room temperature for 5 min, centrifuge at 16,000× *g* for 10 min at 27 °C. Thereafter, remove the aqueous (upper) phase (approximately 400 μL) and transfer to a clean tube.
6. Add 40 μL of 3M KOAc (pH 5.5) and 500 μL of ice-cold isopropanol to the sample and vortex well.
7. Centrifuge at 16,000× *g* for 30 min at 4 °C. Thereafter, aspirate off the supernatant and save the pellet.
8. Add 1 mL of ice-cold 70% (vol/vol) ethanol to the sample.
9. Centrifuge at 16,000× *g* for 5 min at 4 °C. Thereafter, aspirate off the supernatant and save the pellet.
10. Add 40 μL of RNase-free water and dissolve the ribosomal RNA pellet.
11. Mix 0.5 μL of the sample, 4 μL of RNase-free water, and 4.5 μL of 2x RNA loading dye.
12. Apply the resulting sample onto a 4% PAGE (8M urea) gel and run it at 190 V (constant voltage) until the XC dye reaches the bottom of the gel (approximately 1 h).
13. Visualize the RNA bands by EtBr staining.

**Notes**

1. For typical results of the analysis of ribosomal proteins and ribosomal RNAs, refer to [18].
2. When verifying the ribosome activities, it is recommended to simultaneously examine the appropriate Mg^2+^ and polyamine concentration of the reaction, in order to recapitulate polyproline-mediated ribosome stalling; polyproline translation should be analyzed under the Mg^2+^ concentration of 5–7 mM in the presence of 0.25 mM spermidine and 0 mM spermine (see Figure 2). Refer to Section 3.4.3 for the translation reaction method and to Table S4 for the translation reaction conditions.
3. When extracting ribosomal RNAs for analysis, TRIzol LS (Thermo Fisher Scientific) can be used as an alternative to ISOGEN-LS.

#### 3.1.10. tRNAs

**Cell culture**

1. From a glycerol stock, streak a sample of YPH499 yeast cells on a YPD plate and grow at 30 °C for 2 days.
2. Collect the cells from the plate using a disposable loop, inoculate a 300 mL culture of YPD medium in a 500 mL flask with the cells, and then grow overnight (approximately 16 h) at 30 °C with vigorous shaking at 170 rpm.
3. Inoculate six 900 mL cultures of YPD medium in 2 L baffled flasks with 20 mL of the overnight culture per flask, and then, grow at 30 °C with vigorous shaking at 170 rpm, until the culture reaches an OD_600_ of 4.0. This should take approximately 9 h.
4. Harvest the cells by centrifugation at 9000× *g* for 10 min at 4 °C.
5. Resuspend with 1 L of 1% (wt/vol) KCl and harvest the cells by centrifugation at 9000× *g* for 10 min at 4 °C.
6. Resuspend the cells with approximately 100 mL of lysis buffer and divide into six 50 mL tubes.
7. Harvest the cells by centrifugation at 9000× *g* for 10 min at 4 °C. The pellet weight is about 4 g per tube.

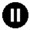
** PAUSE STEP** Frozen cells can be stored at −80 °C for approximately one month.

**Preparation of crude tRNAs**

1. Add 8 mL of 1 mM Tris-HCl (pH 7.5)–10 mM Mg(OAc)_2_ per tube (2 mL per 1 g cell pellet) and resuspend well.
2. Add 4 mL of phenol saturated with 1 mM Tris-HCl (pH 7.5)–10 mM Mg(OAc)_2_ per tube (1 mL per 1 g cell pellets) and vortex well.
3. Shake vigorously using a shaker overnight at 27 °C.
4. Centrifuge the samples at 8500× *g* for 30 min at 27 °C. Thereafter, transfer the aqueous phase to six clean tubes (approximately 10 mL per tube).

**NOTE:** Make sure that the supernatants are clear, in order to avoid DNA contamination.

5. Add 1 mL (1/10 volumes of sample) of 5 M NaCl and 20 mL (2 volumes of sample) of 100% (vol/vol) ethanol per tube, and then, vortex well.
6. Centrifuge the samples at 8500× *g* for 30 min at 27 °C. Thereafter, aspirate off the supernatants.
7. Add gently 10 mL of 100% (vol/vol) ethanol per tube.
8. Centrifuge the samples at 8500× *g* for 5 min at 4 °C. Thereafter, aspirate off the supernatants.
9. Dry the pellets for 5 min at 27 °C.
10. Resuspend the pellets with 5 mL of 1 M NaCl per tube.
11. Centrifuge the samples at 8500× *g* for 30 min at 4 °C. Thereafter, transfer the supernatant to six clean tubes (approximately 5 mL per tube).

**NOTE:** Here, high molecular weight RNAs are selectively precipitated as the pellets.

12. Add 10 mL (2 volumes of sample) of 100% (vol/vol) ethanol per tube and vortex well.
13. Centrifuge the samples at 8500× *g* for 30 min at 4 °C. Thereafter, aspirate off the supernatants.
14. Resuspend the pellets with 8 mL of 1.8 M Tris-HCl (8.0) per tube.
15. Incubate the samples at 37 °C for 1.5 h, in order to deacylate the endogenous aminoacyl-tRNAs.
16. Add 800 μL of 5 M NaCl and 20 mL of 100% (vol/vol) ethanol per tube and vortex well.
17. Centrifuge the samples at 8500× *g* for 30 min at 4 °C. Thereafter, aspirate off the supernatants.

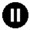
** PAUSE STEP** Freeze the sample and they can be stored at −80 °C for approximately 1 day.

18. Resuspend the pellets with 25 mL of HiTrap Q A buffer.
19. Quantify the crude tRNAs using a spectrometer. The yield is about 800 A_260_ units.

**Purification of tRNAs by HiTrap Q column chromatography**

1. Load the sample at a flow rate of 1.0 mL/min onto a HiTrap Q HP column (15 mL, Cytiva) which has been equilibrated with 0% (200 mM NaCl) Q B buffer.

**NOTE:** no more than 70 A_260_ units of sample should be loaded per 1 mL of resin.

2. After washing with 5 column volumes of 0% (200 mM NaCl) Q B buffer, wash further with a linear gradient from 0 to 20% (200 to 360 mM NaCl) Q B buffer in 2.5 column volumes at a flow rate of 2.0 mL/min.
3. Elute the tRNAs with a linear gradient from 20 to 35% (360 to 480 mM NaCl) Q B buffer in 9 column volumes, with an additional linear gradient from 35 to 45% (480 to 560 mM NaCl) Q B buffer in 7.25 column volumes, at a flow rate of 2.0 mL/min, collecting 3 mL fractions.
4. Wash the column with a linear gradient from 45 to 100% (560 mM to 1 M NaCl) Q B buffer in 1.25 column volumes, and with 1 column volume of 100% (1 M NaCl) Q B buffer.
5. Identify the fractions containing tRNAs by analyzing the aliquots (5 μL) of fractions by 10% PAGE (8M urea) and staining with EtBr. tRNAs are typically eluted from 30 to 40% B buffer. Pool fractions that contain tRNAs; the volume of the pool is approximately 110 mL.

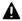
** CRITICAL STEP** Be careful not to collect longer RNAs, including 5S RNA, which elute following the major peak of tRNAs at about 40% B buffer.

6. Add 0.1 volumes of 3 M NaOAc (pH 5.5) and an equal volume of ice-cold isopropanol to the samples and mix well.
7. Allow the sample to cool sufficiently at −30 °C overnight (or in liquid nitrogen for a while).
8. Centrifuge the samples at 7500× *g* for 1 h at 4 °C. Thereafter, aspirate off the supernatants.
9. Resuspend the pellets with 1.5 mL of HiTrap Q A buffer.
10. Add 150 μL (0.1 volumes) of 3 M NaOAc (pH 5.5) and 3 mL (two volumes) of ice-cold ethanol to the samples and mix well. Divide the sample into two 5 mL tubes.
11. Centrifuge the samples at 15,000× *g* for 1 h at 4 °C. Thereafter, aspirate off the supernatants.
12. Add 2 mL of ice-cold 70% (vol/vol) ethanol per tube and vortex well.
13. Centrifuge the samples at 15,000× *g* for 20 min at 4 °C. Thereafter, aspirate off the supernatants. Remove as much ethanol as possible.
14. Dry the pellets at 27 °C for approximately 5 min. Do not over-dry the pellets; they should be slightly wet.
15. Dissolve the pellets with a total of 600 μL RNase-free water.
16. Measure the A_260_ of the sample using a spectrometer, and determine the concentration of tRNAs.
17. The samples are aliquoted, fast-frozen in liquid nitrogen, and stored at −80 °C. The yield is about 120 A_260_ units/L culture (Table S2).

**Analysis of tRNAs**

1. Add 5 μL of 2× RNA loading dye to 0.01 A_260_ units of the sample and mix well.
2. Apply the resulting sample onto a 10% PAGE (8M urea) gel and run it at 200 V (constant voltage) until the BPB dye reaches the bottom of the gel (approximately 40 min).
3. Visualize the tRNA bands by EtBr staining.

**Notes**

1. For typical results of the PAGE analysis of tRNAs, refer to [18].
2. Examine the aminoacylation efficiency of the tRNAs by analyzing the charging of [^35^S]Met, according to the “preparation of aminoacyl-tRNA” section.

### 3.2. Aminoacyl-tRNAs

**Preparation of S100 yeast lysate**
**Cell culture**

1. From a glycerol stock, streak a sample of YPH499 yeast cells on a YPD plate and grow at 30 °C for 2 days.
2. Collect the cells from the plate using a disposable loop, inoculate a 300 mL culture of YPD medium in a 500 mL flask with the cells, and then grow overnight (approximately 16 h) at 30 °C with vigorous shaking at 170 rpm.
3. Inoculate twelve 900 mL cultures of YPD medium in 2 L baffled flasks with 20 mL of the overnight culture per flask, and then, grow at 30 °C with vigorous shaking at 170 rpm, until the culture reaches an OD_600_ of 4.0. This should take approximately 9 h.
4. Harvest the cells by centrifugation at 9000× *g* for 10 min at 4 °C.
5. Resuspend with 1 L of 1% (wt/vol) KCl and harvest the cells by centrifugation at 9000× *g* for 10 min at 4 °C.
6. Resuspend with 1 L of lysis buffer and harvest the cells by centrifugation at 9000× *g* for 10 min at 4 °C. The pellet weight should be 4.0 g/L culture.
7. Resuspend with an equal amount of lysis buffer (1 mL buffer per 1 g of cells) and dispense the cells through micropipettes into liquid nitrogen to form frozen yeast droplets.

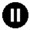
** PAUSE STEP** Frozen cells can be stored at −80 °C for approximately one month.

**Preparation of S100 lysate**

1. Disrupt the cells by mechanical grinding (see Appendix A).
2. Thaw the frozen powder of ground cells at 4 °C and centrifuge at 7500× *g* for 10 min at 4 °C. Thereafter, transfer the supernatants to a clean tube (Sup 1, approximately 40 mL).
3. Resuspend the resulting pellets with 20 mL of lysis buffer and centrifuge at 7500× *g* for 10 min at 4 °C. Thereafter, transfer the supernatants to a clean tube (Sup 2, approximately 20 mL).
4. Combine Sup 1 and Sup 2 and transfer to a clean ultracentrifuge tube. Centrifuge at 20,000× *g* (16,000 rpm, Type 45Ti, BECKMAN COULTER) for 30 min at 4 °C. Thereafter, transfer the supernatants to a clean tube (approximately 50–60 mL).

**NOTE:** Take care to minimize contamination from either the lipid fraction at the very top or the cell debris at the bottom of the tube.

5. Centrifuge the samples at 65,000× *g* (29,000 rpm, Type 45Ti, BECKMAN COULTER) for 5 h at 4 °C. Thereafter, transfer the top two-thirds of the supernatant to a clean tube (approximately 45 mL).

**NOTE:** Take care to minimize contamination from either the lipid fraction at the very top or the cell debris at the bottom of the tube.

6. Dialyze the sample against 2 L of Buffer A at 4 °C overnight, with a change of buffer after 1 h.
7. **OPTIONAL STEP:** Clarify the sample by centrifugation at 10,000× *g* for 15 min at 4 °C.
8. Mix the sample and 10 mL of Q sepharose Fast Flow resin (Cytiva) equilibrated with Buffer A (250 mM NaCl) on a rotator at 4 °C for approximately 30 min.
9. Sediment the resin by centrifugation at 500× *g* for 10 min, and then, transfer the supernatant to a clean tube (flow-through Fraction 1, approximately 40 mL).
10. Add 1 column volume of Buffer A (250 mM NaCl) to the resin and gently mix the slurry. Sediment the resin by centrifugation at 500× *g* for 10 min, and then, transfer the supernatant to a clean tube (flow-through Fraction 2, approximately 10 mL).
11. Combine the flow-through Fractions 1 and 2 (approximately 50 mL).
12. Measure the concentration of the sample by the Bradford Assay.
13. The samples are aliquoted in approximately single-use aliquots (50 μL), fast-frozen in liquid nitrogen, and stored at −80 °C, where they usually remain stable for months. The yield of proteins in the S100 lysate is approximately 30 mg from 1 L culture (Table S2).

**Notes**

1. Measure the aminoacylation activity of S100 lysate according to the “preparation of aminoacyl-tRNAs” section.

**Preparation of aminoacyl-tRNAs**

1. Prepare “buffer mixture” and “enzyme mixture” (total 1 mL) on ice, as follows (Table 1)
2. Mix “buffer mixture” and “enzyme mixture”, and then, incubate at 30 °C for 30 min.
3. Add 3 mL of solution D and 300 μL of 2 M NaOAc (pH 4.0) to the reaction mixture, and then, mix well.
4. Add 4.3 mL of phenol saturated with 50 mM KOAc (pH 5.5) and 860 μL of CIA (chloroform:isoamyl alcohol, 49:1) to the sample, and then, vortex well.
5. Centrifuge the sample at maximum speed (~15,000× *g*) for 5 min at 27 °C. Thereafter, transfer the aqueous (upper) phase to a clean tube.
6. Add 4.3 mL of ice-cold isopropanol to the sample and vortex well.
7. Centrifuge the sample at maximum speed (~15,000× *g*) for 30 min at 4 °C. Thereafter, aspirate off the supernatant.
8. Dry the pellet for 5 min at 27 °C.
9. Resuspend the pellet with 300 μL of ice-cold 50 mM KOAc (pH 5.5).
10. Prepare two NAP-5 columns (Cytiva), and equilibrate each column with 10 mL of ice-cold equilibration buffer (0.3 M KOAc (pH 5.5)—5% (vol/vol) isopropanol).

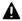
** CRITICAL STEP:** it is recommended that the columns be set in a cold room.

11. Load 150 μL of sample per column and allow the sample to enter the gel bed completely.
12. Load 350 μL of ice-cold equilibration buffer per column, such that combined volume of the sample and equilibration buffer equals 500 μL. Allow the equilibration buffer to enter the gel bed completely.
13. Elute with 560 μL of ice-cold equilibration buffer per column, collecting the sample in a 5 mL tube.
14. Add 2 volumes (1.12 mL) of ice-cold 100% ethanol to the eluate per tube and vortex well.
15. Centrifuge the sample at maximum speed (~15,000× *g*) for 30 min at 4 °C. Thereafter, aspirate off the supernatant.
16. Gently add 2 mL of ice-cold 70% (vol/vol) ethanol per tube, to rinse the pellet.
17. Centrifuge the sample at maximum speed (~15,000× *g*) for 5 min at 4 °C. Thereafter, aspirate off the supernatant. Remove as much ethanol as possible.
18. Dry the pellet for 5 min at 27 °C. Do not over-dry the pellets; they should be slightly wet.
19. Resuspend the pellet with 100 μL of ice-cold 50 mM KOAc (pH 5.5).
20. The aminoacyl-tRNAs are aliquoted in approximately single-use aliquots (60 μL), fast-frozen in liquid nitrogen, and stored at −80 °C, where they usually remain stable for months.

**Evaluation of aminoacylation efficiency by analysis of the [^35^S] Methionine charging**

1. Dilute 1 μL of the aminoacyl-tRNAs 100-fold with 50 mM KOAc (pH 5.5).
2. Spot 1 μL of the resulting sample on Whatman 3MM chromatography paper (1 cm × 1 cm) (Whatman).
3. Immerse the paper in ice-cold 10% (vol/vol) TCA and gently shake for 10 min on ice.
4. Change the ice-cold 10% (vol/vol) TCA and gently shake for 10 min on ice.
5. Immerse the paper in ice-cold 70% (vol/vol) ethanol and gently shake for 10 min on ice.
6. Dry up the paper completely under a heat lamp (approximately 15 min).
7. Transfer the dried paper into a plastic scintillation vial containing 5 mL of Ultima Gold scintillation cocktail (PerkinElmer). In parallel, in order to measure the specific radioactivity of radioisotopic methionine, undiluted [^35^S]methionine stock solution is diluted 10,000-fold with RNase-free water, and 1 μL of the dilution corresponding to 8.5 × 10^−4^ pmol [^35^S]methionine is transferred into another plastic scintillation vial containing 5 mL of Ultima Gold.
8. Vigorously shake the samples for 30 min at 27 °C.
9. Measure the radioactivity of the samples using a scintillation counter.

**Notes**

1. Approximately 8 pmol of [^35^S]methionine is usually charged per one A_260_ unit of tRNA mixture.
2. If the aminoacylation efficiency is inefficient, the volume of S100 in the reaction can be increased up to 1/5 reaction volumes (200 μL).
3. Verify the translation ability of the aminoacyl-tRNAs, referring to Section 3.4.3 for the translation reaction method, and to Table S4 for the translation reaction condition.

### 3.3. Preparation of CrPV-IRES Containing mRNA

Prepare the desired mRNAs by in vitro transcription using T7 RNA polymerase and the template DNAs, according to the standard protocols (for details, see Appendix B). The template DNA sequences for the mRNAs used in Figure 2 are shown in Table S6.

### 3.4. In Vitro Translation

#### 3.4.1. Transcription-Aminoacylation-Translation Coupled Reaction

The protocols in this section describe a standard reaction, where a DNA template and T7 RNA polymerase are included for the mRNA transcription, and human aminoacyl-tRNA synthetase mixture [4] is included for aminoacylation. The Mg^2+^ and polyamine concentration in the reaction, [Mg/SPD/SP], is set as [9/2/0.1], in order to sustain the efficient transcription and aminoacylation reactions. Note that polyproline-mediated translation arrest is not recapitulated under this condition.

1. Prepare the “buffer mixture,” “enzyme mixture,” and “DNA solution” (total 20 μL) on ice, as follows (Table 2):

2. Mix “buffer mixture,” “DNA solution,” and “enzyme mixture,” in this order, on ice.
3. Incubate the reaction mixture at 30 °C for an appropriate time (2–4 h).
4. Put the sample on ice, to stop the reaction, at an appropriate time point.

#### 3.4.2. Aminoacylation-Translation Coupled Reaction

The protocols in this section describe a standard reaction, where human aminoacyl-tRNA synthetase mixture [4] is included for aminoacylation. mRNAs are utilized in the reaction, instead of DNA template and T7 RNA polymerase. The Mg^2+^ and polyamine concentration in the reaction, [Mg/SPD/SP], is set as [9/2/0.1], in order to sustain the efficient aminoacylation reactions. Note that polyproline-mediated translation arrest is not recapitulated under this condition.

1. Prepare the “buffer mixture,” “enzyme mixture,” and “mRNA solution” (total 20 μL) on ice, as follows (Table 3):

2. Mix “buffer mixture,” “enzyme mixture,” and “mRNA solution,” in this order, on ice.
3. Incubate the reaction mixture at 30 °C for an appropriate time (2–4 h).
4. Put the sample on ice, to stop the reaction, at an appropriate time point.

#### 3.4.3. Translation Reaction without Coupled Reaction

The protocols in this section describe a standard reaction where the translation reaction is separated from transcription and aminoacylation reactions; mRNAs are utilized instead of DNA template and T7 RNA polymerase, and a pre-charged aminoacyl-tRNA mixture is used instead of a human aminoacyl-tRNA synthetase mixture [4]. The Mg^2+^ and polyamine concentration in the reaction, [Mg/SPD/SP], is usually set as [5–7/0.25/0], such that polyproline-mediated translation arrest is properly recapitulated. The precise Mg^2+^ concentration needs to be determined, depending on the lot of 80S ribosome preparation (see Figure 2).

1. Prepare the “buffer mixture,” “enzyme mixture,” “aminoacyl-tRNA mixture,” and “mRNA solution” (total 20 μL) on ice, as follows (Table 4):

2. Mix “buffer mixture,” “enzyme mixture,” “mRNA solution,” and “aminoacyl-tRNA mixture,” in this order, on ice.
3. Incubate the reaction mixture at 30 °C for an appropriate time (2–4 h).
4. Put the sample on ice, to stop the reaction, at an appropriate time point.

### 3.5. Analysis of Translation Products

#### 3.5.1. Measurement of Nanoluciferase Activity

1. After the translation reaction, mix 2 μL of the translation reaction sample and 18 μL of 0.1 mg/mL RNase A, to stop the reaction, on ice.
2. Prepare a Nano-Glo Substrate-Buffer mixture, which contains Nano-Glo Luciferase Assay Substrate (Promega) and Nano-Glo Luciferase Assay Buffer (Promega) in a ratio of 1:50. Mix the sample in Step 1 (20 μL) and 20 μL of Nano-Glo Substrate-Buffer mixture on ice.
3. Apply the sample into a white 96-well half-area plate and incubate for 17 min at 27 °C.
4. Measure the nanoluciferase activity using a GloMax platereader (Promega) (PROTOCOLS: Nano-Glo/Integration Time: 0.3 s).

**Notes**

1. When analyzing the time course of the nanoluciferase synthesis, withdraw 2 μL from the reaction solution at the desired time point. At this time, gently open the lid of the reaction tube, and do not remove the tube from the heat block, in order to minimize the temperature change of the sample. Gently aliquot 2 μL from the very top of the reaction solution. Do not disturb the reaction solution by pipetting.

#### 3.5.2. Detection of ^35^S-Met-Labeled Proteins by SDS-PAGE

1. After the translation reaction, mix the translation reaction sample (10 μL) and 0.4 μL of 5 mg/mL RNase A, and then, incubate for 5 min at 30 °C. Thereafter, add 2.5 μL of 5x LiDS dye to the sample and mix well.
2. Apply the resulting sample onto a 15% (or 18%) SDS-PAGE gel and run it at 190 V (constant voltage) for approximately 2 h.
3. Immerse the gel in 10% (vol/vol) acetic acid—10% (vol/vol) ethanol and shake at room temperature for 30 min.
4. Repeat Step 3.
5. Immerse the gel in 10% (vol/vol) acetic acid—40% (vol/vol) ethanol—3% (vol/vol) glycerol, and then, shake at room temperature for 1 h.
6. Transfer the gel onto a piece of filter paper, place plastic wrap over the gel, and dry with a gel dryer.
7. Expose the gel on an imaging plate for an appropriate time (approximately 6 h) and analyze using a Bio-Analyzing System (BAS5000, Fujifilm). A filter paper spotted with a dilution series of [^35^S] Met (4.2 × 10^−4^–6.8 × 10^−2^ pmol) should be also analyzed, in order to obtain a standard curve for [^35^S] Met.

**Notes**

1. When analyzing peptidyl-tRNAs, the sample is directly mixed with 5× LiDS dye, without adding RNase A, and subjected to SDS-PAGE analysis.
2. When analyzing short peptides (<10 kDa), Tricine SDS-PAGE analysis is recommended.

#### 3.5.3. Analysis of Incorporation of ^35^S-Met into Oligopeptides

1. After the translation reaction, mix the translation reaction sample (10 μL) and 10 μL of 1 M HCl, and then, vortex for 30 s.
2. Add 200 μL of ice-cold ethyl acetate to the sample and vortex for 60 s.

**NOTE:** Tighten the tube lid and do not warm the tube with your fingers. Otherwise, the sample may leak from the tube during vortexing.

3. Keep the sample on ice for 10 min.
4. Centrifuge the sample at maximum speed (~15,000× *g*) for 5 min at 4 °C. Thereafter, transfer the supernatants (~160 μL) to a plastic vial containing 5 mL of Ultima Gold (Perkin Elmer).
5. Shake the sample vigorously for 10 min at room temperature.
6. Measure the radioactivity of the sample with a scintillation counter.

## 4. Expected Results

Figure 2 shows the analysis of the translation activity of the prepared ribosomes, where the translation of the polyproline sequence, in response to the Mg^2+^ concentration and the effect of eIF5A, was examined. Two types of reporter mRNA encoding for nanoluciferase were prepared: no-motif and Prox4. In Prox4 mRNA, the polyproline sequence is inserted in front of the nanoluciferase sequence, while there is no insertion sequence at the corresponding position in no-motif mRNA (Figure 2A, Table S6). These two reporter mRNAs were translated in the presence and absence of eIF5A at Mg^2+^ concentrations of 5, 6, and 7 mM, and the nanoluciferase activities were measured (Figure 2B). With the ribosomes of this preparation, we consider that the appropriate condition for further studies is 6 mM Mg^2+^, for the following reasons: (i) polyproline-mediated translation arrest was properly observed (Figure 2B, Panel 5), and (ii) the translation efficiency of the no-motif mRNA in the absence and presence of eIF5A was approximately the same at a reaction time of 120 min (Figure 2B, Panel 2). Note that, under the 7 mM Mg^2+^ condition, polyproline-mediated translation arrest was no longer observed (Figure 2B, Panel 6). Moreover, translation of no-motif mRNA was considerably inhibited by eIF5A (Figure 2B, Panel 3). The reason for this is still unclear; one possibility is that eIF5A promotes premature termination at several sites in the ORF (for a detailed discussion, see [24]). On the other hand, under the 5 mM Mg^2+^ condition, the translation initiation and the early elongation process were presumably inefficient in the absence of eIF5A (Figure 2B, Panel 1). Consequently, the effect of eIF5A on the translation of the polyproline sequence, per se, would not be appropriately evaluated under such a condition (Figure 2B, Panel 4). Figure 2C shows the analysis of the translation products at 6 mM Mg^2+^ condition by Tricine SDS-PAGE. In agreement with the nanoluciferase activities, the truncated proteins are observed from the Prox4 mRNAs, and the addition of eIF5A stimulates the production of full-length nanoLuciferase.

For other examples of detailed analyses of translation elongation regulation with the reconstituted yeast translation system, see [24]. Ribosome stalling by the polyproline sequence in either oligopeptides or long polypeptides, CGA codon repeats, and the polytryptophan sequence have been analyzed using the methods described herein. The effect of Mg^2+^/polyamine on the translation of the polyproline sequence has also been presented in detail.

## Figures and Tables

**Table 1:** Components in the aminoacylation reaction.

	Components	Volume (μL)
Buffer mixture	1.5 mM 18 a.a (without Met and Cys)	100
	10 mM Cysteine	10
	tRNAs from yeast	(6.25 A_260_ units)
	10× NTPs	100
	12% PURE buffer (without Mg and SPD)	120
	1 M Creatine phosphate	20
	500 mM Mg(OAc)_2_	X (final 9 mM) *^1^
	20 mM spermidine	100
	10 mM spermine	10
	[^35^S]methionine *^2^	50
Enzyme mixture	RNase-free water	(to 1 mL)
	Creatine kinase	(final 100 nM)
	Myokinase	(final 20 nM)
	Nucleoside-diphosphate kinase	(final 15 nM)
	Pyrophosphatase	(final 15 nM)
	eEF1A	(final 0.5 μM)
	Recombinant RNase Inhibitor	20
	S100	50

*^1^ The components listed in Table S5 are dissolved in stock buffer containing the indicated Mg^2+^ concentrations [1,4,18]. X is determined by taking into account the concentration of Mg^2+^ derived from the components, such that the final concentration of Mg^2+^ in the reaction is 9 mM. *^2^ When preparing the unlabeled aminoacyl-tRNAs, add 50 μL of 1 mM methionine, instead of [^35^S]methionine.

**Table 2:** Components in the “Transcription-Aminoacylation-Translation Coupled Reaction”.

	Components	Volume (μL)
Buffer mixture	1.5 mM 18 a.a. (without Met and Cys)	2
	10 mM Cys	0.2
	Yeast tRNAs	(0.125 A_260_ units)
	10× NTPs	2
	12% PURE buffer (without Mg and SPD)	2.4
	1 M Creatine phosphate	0.4
	250 mM Mg(OAc)_2_	X *^1^ (final 9 mM)
	20 mM SPD	2
	10 mM SP	0.2
	1 mM Met *^2^	1
Enzyme mixture	T7 RNA polymerase	(final 30 nM)
	Creatine kinase	(final 100 nM)
	Myokinase	(final 20 nM)
	Nucleoside-diphosphate kinase	(final 15 nM)
	Pyrophosphatase	(final 15 nM)
	eEF1A	(final 5.0 μM)
	eEF2	(final 0.5 μM)
	eEF3	(final 0.5 μM)
	eRF1	(final 0.5 μM)
	eRF3	(final 0.5 μM)
	Dom34	(final 0.5 μM)
	Hbs1	(final 0.5 μM)
	Rli1	(final 0.5 μM)
	80S ribosome	(final 0.5 μM)
	ARS^human^ mix	(final 0.05 mg/mL)
DNA solution	Template DNA	(final 0.1 nM)
	RNase-free water	(to 20 μL)

*^1^ The components listed in Table S5 are dissolved in stock buffer containing the indicated Mg^2+^ concentrations [1,4,18]. X is determined by taking into account the concentration of Mg^2+^ derived from the components, such that the final concentration of Mg^2+^ in the reaction is 9 mM. *^2^ When analyzing the [^35^S]methionine-labelled translation products, add 1 μL of [^35^S]methionine (PerkinElmer, NEG009A) instead of cold methionine.

**Table 3:** Components in the “Aminoacylation-Translation Coupled Reaction”.

	Components	Volume (μL)
Buffer mixture	1.5 mM 18 a.a. (without Met and Cys)	2
	10 mM Cys	0.2
	Yeast tRNAs	(0.125 A_260_ units)
	20 mM ATP, GTP	1
	12% PURE buffer (without Mg and SPD)	2.4
	1 M Creatine phosphate	0.4
	250 mM Mg(OAc)_2_	X *^1^ (final 9 mM)
	20 mM SPD	2
	10 mM SP	0.2
	1 mM Met *^2^	1
Enzyme mixture	Creatine kinase	(final 100 nM)
	Myokinase	(final 20 nM)
	Nucleoside-diphosphate kinase	(final 15 nM)
	Pyrophosphatase	(final 15 nM)
	eEF1A	(final 5.0 μM)
	eEF2	(final 0.5 μM)
	eEF3	(final 0.5 μM)
	eRF1	(final 0.5 μM)
	eRF3	(final 0.5 μM)
	Dom34	(final 0.5 μM)
	Hbs1	(final 0.5 μM)
	Rli1	(final 0.5 μM)
	80S ribosome	(final 0.5 μM)
	ARS^human^ mix	(final 0.05 mg/mL)
mRNA solution	mRNA	(final 0.25–0.5 μM)
	RNase-free water	(to 20 μL)

*^1^ The components listed in Table S5 are dissolved in stock buffer containing the indicated Mg^2+^ concentrations [1, 4, 18]. X is determined by taking into account the concentration of Mg^2+^ derived from the components, such that the final concentration of Mg^2+^ in the reaction is 9 mM. *^2^ When analyzing the [^35^S]methionine-labelled translation products, add 1 μL of [^35^S]methionine (PerkinElmer, NEG009A) instead of cold methionine.

**Table 4:** Components in the “Translation Reaction without Coupled Reaction”.

	Components	Volume (μL)
Buffer mixture	1.5 mM 18 a.a. (without Met and Cys)	2
	10 mM Cys	0.2
	20 mM ATP, GTP	1
	12% PURE buffer (without Mg and SPD)	2.4
	1 M Creatine phosphate	0.4
	250 mM Mg(OAc)_2_	X *^1^
	20 mM SPD	0.25
	1 mM Met *^2^	1
Enzyme mixture	Creatine kinase	(final 100 nM)
	Myokinase	(final 20 nM)
	Nucleoside-diphosphate kinase	(final 15 nM)
	Pyrophosphatase	(final 15 nM)
	eEF1A	(final 5.0 μM)
	eEF2	(final 0.5 μM)
	eEF3	(final 0.5 μM)
	eRF1	(final 0.5 μM)
	eRF3	(final 0.5 μM)
	Dom34	(final 0.5 μM)
	Hbs1	(final 0.5 μM)
	Rli1	(final 0.5 μM)
	80S ribosome	(final 0.5 μM)
mRNA solution	mRNA	(final 0.25–0.5 μM)
	RNase-free water	(to 20 μL)
Aminoacyl-tRNA mixture	Aminoacyl-tRNA	(0.125 A_260_ units)

*^1^ The components listed in Table S5 are dissolved in stock buffer containing the indicated Mg^2+^ concentrations [1,4,18]. X is determined by taking into account the concentration of Mg^2+^ derived from the components, such that the final concentration of Mg^2+^ in the reaction is the desired concentration (usually 5–7 mM). *^2^ When analyzing the [^35^S]methionine-labelled translation products, add 1 μL of [^35^S]methionine (PerkinElmer, NEG009A), instead of cold methionine.

## Data Availability

Not applicable.

## Supplementary Materials

The following are available online at https://www.mdpi.com/article/10.3390/mps4030045/s1, Table S1. Summary of the experimentally estimated absorption coefficients of the purified factors, Table S2. Summary of the expression and purification of the components for the reconstituted translation system, Table S3. Yeast strains used for the purification of the components for the reconstituted translation system, Table S4. Translation reaction condition for checking the activity of the components in the reconstituted translation system, Table S5. Components in the reconstituted translation system dissolved in buffer that contained Mg^2+^, Table S6. Sequence of template DNA.

## Appendix A. Cell lysis

**Disruption of yeast cells by mechanical grinding**

1. Fill the grinder (Retsch, Mortar Grinder RM200) with liquid nitrogen and preliminarily run the mortar before adding frozen cells.

** CRITICAL STEP** Pre-cool the mortar and pestle with liquid nitrogen before cell lysis, in order to prevent frozen cells from melting. We recommend pre-running the grinder fully filled with liquid nitrogen more than three times, until boiling of the liquid nitrogen stops.

2. Pour liquid nitrogen and transfer 15 g of frozen cells (30 g of yeast drops) into the mortar, and grind them for 2–3 min.
3. Repeat step 2 about five times (15 min in total).
4. Transfer the frozen powder to tubes with a pre-chilled spatula.

**Disruption of *E. coli* cells by sonication**

1. Resuspend cells with 5 mL of ice-cold lysis buffer per 1 g of cells.
2. The mixture is transferred to an 100 mL aluminum container and placed on ice.

** CRITICAL STEP** The volume of the sample should not exceed 75 mL for efficient disruption.

3. Disrupt the cells using a sonicator (Branson, Digital Sonifier 450) (Run time: 5 min, Pulse on: 2 s, Pulse off: 8 s, Power: 80 watt), until the lysate becomes clear.

## Appendix B. Preparation of mRNA

**Preparation of template DNA by PCR**

Prepare the PCR mixture (200 μL) in 0.2 mL tubes, as follows: Mix 12 μL of 25 mM MgSO_4_, 20 μL of 10× PCR Buffer for KOD-Plus-Neo, 20 μL of 2 mM dNTPs, 6 μL of 10 μM forward primer, 6 μL of 10 μM reverse primer, 100 ng of chemically synthesized DNA fragments (Table S5) or plasmid harboring the desired sequence, 4 μL of KOD-Plus-Neo (Toyobo), and RNase-free water to 200 μL. Run the sample in a thermal cycler with the following program: 94 °C for 2 min, and 30 cycles of 98 °C for 10 s, 57 °C for 15 s, and 68 °C for 30 s per 1 kb amplified sequence. After PCR, add 4 μL of *Dpn* I to the sample (200 μL) and incubate at 37 °C for 1 h. Purify the sample using Wizard SV Gel and PCR Clean-Up System (Promega), according to the manufacturer’s instructions. Measure the concentration of the PCR product using a spectrometer and check the amplified DNA templates using agarose gel electrophoresis. The DNA templates are aliquoted to minimize freeze-thawing, and stored at −30 °C.

**Analysis of template DNA by agarose gel electrophoresis**

Mix 0.1 volumes of 10× Loading Buffer (Takara) and 1000 ng of the template DNAs by PCR. Apply the resulting sample onto 1% agarose gel and run it with 0.5× TAE at 100 V for 30–40 min. Stain the gel with 1/20,000-diluted EtBr solution with gentle shaking for 30 min. Scan the gel image with a gel scanner.

**In vitro transcription of mRNAs**

Prepare the reaction mixture (100 μL) in a microtube, as follows: Mix 10 μL of 10× T7 buffer, 3.75 μL of 100 mM ATP, 3.75 μL of 100 mM GTP, 3.75 μL of 100 mM CTP, 3.75 μL of 100 mM UTP, 5 μL of 100 mM DTT, 10 μL of 0.1% (wt/vol) BSA (TAKARA), 2.5 μL of Recombinant RNase Inhibitor (Takara), 1650 ng of template DNA, pyrophosphatase (100 μg/mL final), T7 RNA polymerase (120 μg/mL final), and RNase-free water to 100 μL. Incubate the reaction mixture at 37 °C for 4 h. Add 1 μL of TURBO DNase (Thermo Fisher Scientific) to the sample (100 μL) and incubate at 37 °C for 15 min. After the reaction, add 35 μL of RNase-free water and 14 μL (1/10 volumes) of 5 M ammonium acetate–100 mM EDTA into the mixture, in order to quench the reaction. Add 150 μL (1 volume) of phenol saturated with H_2_O into the mixture and vortex well. Centrifuge the mixture at 20,000 g for 5 min at 27 °C. Thereafter, transfer the supernatant to a clean tube. Add 150 μL (1 volume) of chloroform into the sample and vortex well. Centrifuge the mixture at 20,000 g for 5 min at 27 °C. Thereafter, transfer the supernatant to a clean tube. Add 300 μL (2 volumes) of ice-cold 100% (vol/vol) ethanol into the sample and vortex well. Centrifuge the mixture at 20,000 g for 15 min at 4 °C. Thereafter, aspirate off the supernatant. Gently add 500 μL of ice-cold 70% (vol/vol) ethanol to the pellet, to rinse. Centrifuge the sample at 20,000 g for 5 min at 4 °C. Thereafter, aspirate off the supernatant. Dry the pellet for 5 min at 27 °C. Add 37.5 μL of RNase-free water and dissolve the pellet. The sample is further purified using Micro Bio-Spin columns P30 (Bio-Rad) to remove NTPs, according to the manufacturer’s instructions. Measure the concentration of the mRNA using a spectrometer and check the mRNA using denaturing agarose gel electrophoresis. The mRNAs are aliquoted, to minimize freeze-thawing, and stored at −80 °C.

**Analysis of mRNAs by denaturing agarose gel electrophoresis**

Mix 500 ng of mRNA, 0.5 μL of 20× MOPS running buffer (pH 7.0), 3.5 μL of formaldehyde, 10 μL of formamide, 1 μL of 1 mg/mL EtBr, and RNase-free water to 20 μL. Incubate the mixture for 10 min at 65 °C. Add 2 μL of 10× RNA loading dye to the sample (20 μL). Apply the resulting sample onto a 1% agarose/formaldehyde gel and run it with 1× MOPS running buffer at 100 V for 30 min. Scan the gel image with a gel scanner.

**Reagents set-up**

**50× TAE:** Mix 242 g of Tris, 57.1 mL of acetic acid, and 100 mL of 0.5 M EDTA (pH 8.0), then add RNase-free water up to 1 L. Then, autoclave it. Can be stored at room temperature.

**0.5× TAE:** Mix 990 mL of RNase-free water and 10 mL of 50× TAE. Can be stored at room temperature.

**1% Agarose gel:** Mix 200 mL of 0.5× TAE and 2 g of agarose (1% (wt/vol) final), and then, microwave it until the agarose dissolves completely. Cool the solution to about 60 °C and pour into a gel tray with the well comb in place.

**10× T7 buffer:** 400 mM Tris-HCl (pH 8.0), 220 mM MgCl_2_, and 10 mM spermidine. Mix 20 mL of 1 M Tris-HCl (pH 8.0), 11 mL of 1 M MgCl_2_, and 500 μL of 1 M spermidine, then add RNase-free water up to 50 mL. Can be stored at −30 °C.

**5 M ammonium acetate-100 mM EDTA:** Mix 9.64 g of ammonium acetate and 5 mL of 0.5 M EDTA (pH 8.0), then add RNase-free water up to 25 mL.

**20× MOPS running buffer (pH 7.0):** 400 mM MOPS-NaOH (pH 7.0), 100 mM NaOAc, and 20 mM EDTA. Mix 41.9 g of MOPS, 4.10 g of NaOAc (anhydrous), and 3.72 g of EDTA∙2Na, then add RNase-free water up to 400 mL. Add 1 M NaOH (approximately 90 mL) into the solution until the pH reaches 7.0. After pH adjustment, add RNase-free water up to 500 mL. Then, autoclave it. Can be stored away from light at room temperature.

**1× MOPS running buffer (pH 7.0):** Mix 380 mL of RNase-free water and 20 mL of 20× MOPS running buffer (pH 7.0). Prepare fresh before use.

**1% Agarose/formaldehyde gel:** Mix 67.2 mL of RNase-free water, 3.75 mL of 20× MOPS running buffer (pH 7.0), and 0.75 g of agarose (1% (wt/vol) final), then microwave it until the agarose dissolves completely. After the solution has cooled down to about 60 °C, add 4.12 mL of formaldehyde using a magnetic stirrer. This operation should be performed in a draft, in order to avoid inhalation of formaldehyde. Set a gel tray with the well comb in place in the draft, and pour the solution into the tray.

**10× RNA loading dye:** 0.25% (wt/vol) bromophenol blue (BPB), 0.25% (wt/vol) Xylene cyanole FF (XC), 2.5 mM EDTA, and 7 M urea. Mix 25 mg of BPB, 25 mg of XC, 50 μL of 0.5 M EDTA (pH 8.0), and 4.2 g of urea, then add RNase-free water up to 10 mL. Can be stored at −30 °C.

## Appendix C. Preparation of eIF5A

The hypusinated eIF5A protein, eIF5A(hyp), is prepared according to [24]. The eIF5A_DHS_DOHH/pET29b plasmid is used for the co-expression of non-tagged eIF5A with Dys1 and Lia1 in *E. coli*. The plasmid is transformed into *E. coli* Rosetta(DE3)pLysS. The cells are grown at 37°C to an OD_600_ of 0.5, and protein expression is induced with 1 mM IPTG at 18°C overnight. The cells are resuspended in a five-fold volume of Buffer A (50 mM Tris-KOAc [pH 7.0], 100 mM KCl, 0.1 mM EDTA, 1 mM DTT) and lysed by sonication. After centrifugation at 25,000 x g for 15 min, the supernatant is collected. The proteins are subjected to HiPrep Q anion exchange chromatography (GE Healthcare), in 50 mM Tris-KOAc [pH 7.0], 100–500 mM KCl, 0.1 mM EDTA, and 1 mM DTT. Typically, eIF5A is eluted at about 450 mM KCl. The eluted fraction is dialyzed against Buffer A. The protein is further purified by HiTrap SP HP chromatography (GE Healthcare), in 50 mM Tris-KOAc [pH 7.0], 100–500 mM KCl, 0.1 mM EDTA, and 1 mM DTT. eIF5A is usually eluted at about 440 mM KCl. The eluted fraction is dialyzed against stock buffer (20 mM Hepes-KOH [pH 7.6], 100 mM KCl, 10% glycerol, 7 mM 2-mercaptoethanol), concentrated to 5 mg/mL, and frozen in small aliquots at −80°C. Yield of eIF5A(hyp) from 1L culture is approximately 14.3 mg. Full hypusine modification of the obtained eIF5A has been confirmed by mass spectrometric analysis.
