# Supplementary material for: In Vitro Reconstitution of Yeast Translation System Capable of Synthesizing Long Polypeptide and Recapitulating Programmed Ribosome Stalling
Source: Methods Protoc. 2021 Jul 4;4(3):45. doi: 10.3390/mps4030045 (PMC8293373; doi:10.3390/mps4030045)
Supplement: Supplementary file 1 [file mps-04-00045-s001.zip › mps-1262037-supplementary.pdf]

Table S1. Summary of the experimentally estimated absorption coefficients of the purified factors. After the absorption spectrum of the factor was measured using a spectrometer and the protein concentration of the factor was determined by the Bradford Assay, the absorption coefficient of the factor at 280 nm at 1 mg/mL was estimated.

Component	A_{280} ($A_{280-320}$)	Dilution for spectrum analysis	A_{280} of undiluted protein	Protein concentration (mg/mL)	Absorption coefficient (@ 1mg/mL)
**eEF1A**	0.805	4	3.2	4.67	0.69
**eEF2**	0.554	4	2.2	2.50	0.89
**eEF3**	1.092	20	22	28.3	0.77
**eRF1**	0.791	5	4.0	6.65	0.59
**eRF3Δ165**	1.052	10	11	14.4	0.73
**Dom34**	0.410	4	1.6	2.16	0.76
**Hbs1**	1.089	4	4.4	4.14	1.1
**Rli1**	0.279	2	0.56	1.47	0.38

Table S2. Summary of the expression and purification of the components for the reconstituted translation system.

Component	Gene	MW (kDa)	Tag (N- or C-terminal)	Strain	Vector	Yield (from 1 L culture)
**eEF1A**	TEF1	50	None	Yeast YPH499		1 mg
**eEF2**	EFT2	93	His-tag (C)	Yeast TKY675		40 mg
**eEF3**	YEF3	116	His-tag (C)	*E. coli* JM109(DE3) pRARE (Promega) Rosetta(DE3)pLysS can be substitued.	pET29b-eEF3 (Hayashi, H., 2018, *J. Biochem.*)	700 mg
**eRF1**	SUP45	49	His-tag (C)	*E. coli* Rosetta(DE3)pLysS (Sigma-Aldrich)	pET21a-eRF1 (Kurata, S., 2013, *Nucleic Acids Res.*)	8.5 mg
**eRF3 165**	SUP35	58	His-tag (C)	*E. coli* BL21(DE3) (Sigma-Aldrich)	pET21a-eRF3 165 (Kurata, S., 2013, *Nucleic Acids Res.*)	24 mg
**Dom34**	DOM34	44	His-tag (C)	*E. coli* Rosetta(DE3)pLysS (Sigma-Aldrich)	pET29b-Dom34 (Abe, T., 2020, *J. Biochem.*)	2.2 mg
**Hbs1**	HBS1	68	His-tag (C)	*E. coli* BL21(DE3) (Sigma-Aldrich)	pETDuet-1-Hbs1 (Abe, T., 2020, *J. Biochem.*)	1.6 mg
**Rli1**	RLI1	68	His-tag (C)	Yeast INVSc1	pYES2-Rli1 (Abe, T., 2020, *J. Biochem.*)	35 mg
**80S ribosome**			-	Yeast W303		110 pmol
**tRNA mixture**			-	Yeast YPH499		120 A_{260} units

Table S3. Yeast strains used for the purification of the components for the reconstituted translation system.

Name	Genotype	Source
**YPH499**	MAT**a** *ura3-52 lys2-801_amber ade2-101_ochre trp1-Δ63 his3-Δ200 leu2-Δ1*	Lab stock
**TKY675**	MAT**a** *ade2 leu2 ura3 his 3 leu2 trp1 eft1::HIS3 ef2::TRP1 pEFT2-His$_6$ LEU2 CEN*	Ortiz, P., 2006, *J. Biol. Chem.*
**INVSc1**	MAT**a** *his3Δ1 leu2 trp1-289 ura3-52*/MAT**α** *his3Δ1 leu2 trp1-289 ura3-52*	Thermofisher Scientific
**W303**	MAT**a**/MAT**α** *{leu2-3,112 trp1-1 can1-100 ura3-1 ade2-1 his3-11,15} [phi+]*	Lab stock

Table S4. Translation reaction condition for checking the activity of the components in the reconstituted translation system.

Component	[Mg^{2+}/Spd/Sp]	mRNA	Note
**eEF1A**	[5/0.25/0]	no motif	eEF3 dependency should be checked.
**eEF2**	[5/0.25/0]	no motif	
**eEF3**	[5/0.25/0]	no motif	
**eRF1**	[5/0.25/0]	no motif	
**eRF3Δ165**	[5/0.25/0]	no motif	
**Dom34**	[5/0.25/0]	no motif	
**Hbs1**	[5/0.25/0]	no motif	
**Rli1**	[5/0.25/0]	no motif	
**80S ribosome**	[5/0.25/0]<br>[6/0.25/0]<br>[7/0.25/0]	no motif<br>Pro x4	Polyproline-mediated ribosomal stalling and its alleviation by eIF5A should be checked.
**Aminoacyl-tRNAs**	[5/0.25/0]	no motif	

Table S5. Components in the reconstituted translation system dissolved in buffer that contained Mg^{2+}.

Component	Mg^{2+} in the stock buffer [mM]	reference
**Creatine kinase**	10	[1]
**Myokinase**	10	[1]
**Nucleoside-diphosphate kinase**	10	[1]
**Pyrophosphatase**	10	[1]
**eEF1A**	5	[18]
**eEF2**	0	[18]
**eEF3**	0	[18]
**eRF1**	0	[18]
**eRF3Δ165**	1	[18]
**Dom34**	0	[18]
**Hbs1**	1	[18]
**Rli1**	0	[18]
**80S ribosome**	5	[18]

Table S6. Sequence of template DNA. The template DNA sequences for the model mRNAs encoding nanoluciferase used in Figure 2 are shown. Translation is initiated from the first codon GCT(Ala) just downstream of the CrPV IRES sequence, which is derived from the native CrPV IRES genome sequence. We note that mutations of the GCT(Ala) codon in these model mRNAs do not affect the translation of nanoluciferase, consistent with the previous study [20]. We also note that the model mRNA constructs lack a 3′ untranslated region (3′ UTR), and the addition of the poly(A)$_{36}$ sequence does not show an obvious effect, at least in terms of the translation of nanoLuciferase under our conditions (unpublished results).

**FLAG-Pgk-nLuc-HA_ [-] (for no no motif mRNA)**
5'-GGGCCTAATACGACTCACTATAGGGAGACCGGAATTCAAAGCAAAAATGTGATCT<br>TGCTTGTAAATACAATTTTGAGAGGTTAATAAATTACAAGTAGTGCTATTTTTGTATTT<br>AGGTTAGCTATTTAGCTTTACGTTCCAGGATGCCTAGTGGCAGCCCCACAATATCCAG<br>GAAGCCCTCTCTGCGGTTTTTCAGATTAGGTAGTCGAAAAACCTAAGAAATTTACCT<br>gctatggattacaaggacgacgacgacaagGAATTATCTTCAAAGTTGTCTGTCCAAGATTTGGACT<br>TGAAGGACAAGCGTGTCTTCATCAGAGTTGACTTCAACGTCCCATTGGACGGTAAG<br>AAGATCACTTCT**ACGGTTTTCACCTTGGAAGATTTCGTTGGTGATTGGAGACAAA**<br>**CTGCTGGTTACAATTTGGATCAAGTCTTGGAACAAGGTGGTGTCTCTTCTTTGTT**<br>**TCAAAACTTGGGTGTTTCCGTTACCCCAATCCAAAGAATAGTTTTGTCTGGTGAA**<br>**AACGGTTTGAAGATCGATATCCATGTTATCATCCCATACGAAGGTTTGTCAGGTG**<br>**ATCAAATGGGTCAAATCGAAAAGATCTTCAAGGTTGTTTACCCAGTTGATGATC**<br>**ACCACTTTAAGGTTATCTTGCACTACGGTACTTTGGTCATTGATGGTGTTACTCC**<br>**AAACATGATCGATTACTTTGGTAGACCTTACGAAGGTATTGCTGTTTTCGATGGT**<br>**AAGAAGATTACTGTCACTGGTACTTTGTGGAACGGTAACAAAATTATCGACGAA**<br>**AGATTGATCAACCCAGACGGTTCTTTGTTGTTCAGAGTTACTATTAACGGTGTTA**<br>**CCGGTTGGAGATTGTGCGAAAGAATTTTGGCT**tacccatacgacgtcccagactacgcgTAA-3'<br><br>(Boxed G, T7 start; single underline, CrPV IRES; small letters, FLAG- or HA-tag; do uble underline, pgk; bold, nLuc)<br><br>Translation of ORF:<br>AMDYKDDDDKELSSKLSVQDLDLKDKRVFIRVDFNVPLDGKKITSTVFTLEDFVGDWRQ<br>TAGYNLDQVLEQGGVSSLFQNLGVSVTPIQRIVLSGENGLKIDIHVIIPYEGLSGDQMGQI<br>EKIFKVVYPVDDHHFKVILHYGTLVIDGVTPNMIDYFGRPYEGIAVFDGKKITVTGTLWN<br>GNKIIDERLINPDGSLLFRVTINGVTGWRLCERILAYPYDVPDYA
**FLAG-Pgk-nLuc-HA_ P[Pro x4] (for Pro x4 mRNA)**

5'-GGGCCTAATACGACTCACTATAGGGAGACCGGAATTCAAAGCAAAAATGTGATCT
TGCTTGTAAATACAATTTTGAGAGGTTAATAAATTACAAGTAGTGCTATTTTTGTATTT
AGGTTAGCTATTTAGCTTTACGTTCCAGGATGCCTAGTGGCAGCCCCACAATATCCAG
GAAGCCCTCTCTGCGGTTTTTCAGATTAGGTAGTCGAAAAACCTAAGAAATTTACCT
gctatggattacaaggacgacgacgacaagGAATTATCTTCAAAGTTGTCTGTCCAAGATTTGGACT
TGAAGGACAAGCGTGTCTTCATCAGAGTTGACTTCAACGTCCCATTGGACGGTAAG
AAGATCACTTCTCCTCCCCCACCG**ACGGTTTTCACCTTGGAAGATTTCGTTGGTG**
ATTGGAGACAAACTGCTGGTTACAATTTGGATCAAGTCTTGGAACAAGGTGGTG
TCTCTTCTTTGTTTCAAAACTTGGGTGTTTCCGTTACCCCAATCCAAAGAATAGTT
TTGTCTGGTGAAAACGGTTTGAAGATCGATATCCATGTTATCATCCCATACGAAG
GTTTGTCAGGTGATCAAATGGGTCAAATCGAAAAGATCTTCAAGGTTGTTTACC
CAGTTGATGATCACCACTTTAAGGTTATCTTGCACTACGGTACTTTGGTCATTGA
TGGTGTTACTCCAAACATGATCGATTACTTTGGTAGACCTTACGAAGGTATTGCT
GTTTTCGATGGTAAGAAGATTACTGTCACTGGTACTTTGTGGAACGGTAACAAA
ATTATCGACGAAAGATTGATCAACCCAGACGGTTCTTTGTTGTTCAGAGTTACTA
TTAACGGTGTTACCGGTTGGAGATTGTGCGAAAGAATTTTGGCTtacccatacgacgtccc
agactacgcgTAA-3'

(Boxed G, T7 start; single underline, CrPV IRES; small letters, FLAG- or HA-tag; do
uble underline, pgk;
gray-shaded, polyproline motif; bold, nLuc)

Translation of ORF:
AMDYKDDDDKELSSKLSVQDLDLKDKRVFIRVDFNVPLDGKKITSPPPPTVFTLEDFVGD
WRQTAGYNLDQVLEQGGVSSLFQNLGVSVTPIQRIVLSGENGLKIDIHVIIPYEGLSGDQ
MGQIEKIFKVVYPVDDHHFKVILHYGTLVIDGVTPNMIDYFGRPYEGIAVFDGKKITVTGT
LWNGNKIIDERLINPDGSLLFRVTINGVTGWRLCERILAYPYDVPDYA
